# High-affinity T cell receptor ImmTAC® bispecific efficiently redirects T cells to kill tumor cells expressing the cancer–testis antigen PRAME

**DOI:** 10.1093/immadv/ltae008

**Published:** 2024-11-02

**Authors:** Ana R Ribeiro, Camille Britton-Rivet, Laura Collins, Ricardo J Carreira, Sylvie Moureau, Adel Benlahrech, Sarah Stanhope, Stephen Harper, Nathaniel Liddy, Tara M Mahon, Kristina Petrovic, Mark Fife, David Depoil, Philip Addis, Nicole Bedke, Lucie Bouard, Ronan O’Dwyer, Duncan Gascoyne, Koustubh Ranade

**Affiliations:** Immunocore Limited, Abingdon, Oxfordshire, United Kingdom; Immunocore Limited, Abingdon, Oxfordshire, United Kingdom; Immunocore Limited, Abingdon, Oxfordshire, United Kingdom; Immunocore Limited, Abingdon, Oxfordshire, United Kingdom; Immunocore Limited, Abingdon, Oxfordshire, United Kingdom; Immunocore Limited, Abingdon, Oxfordshire, United Kingdom; Immunocore Limited, Abingdon, Oxfordshire, United Kingdom; Immunocore Limited, Abingdon, Oxfordshire, United Kingdom; Immunocore Limited, Abingdon, Oxfordshire, United Kingdom; Immunocore Limited, Abingdon, Oxfordshire, United Kingdom; Immunocore Limited, Abingdon, Oxfordshire, United Kingdom; Immunocore Limited, Abingdon, Oxfordshire, United Kingdom; Immunocore Limited, Abingdon, Oxfordshire, United Kingdom; Immunocore Limited, Abingdon, Oxfordshire, United Kingdom; Immunocore Limited, Abingdon, Oxfordshire, United Kingdom; Immunocore Limited, Abingdon, Oxfordshire, United Kingdom; Immunocore Limited, Abingdon, Oxfordshire, United Kingdom; Immunocore Limited, Abingdon, Oxfordshire, United Kingdom; Immunocore Limited, Abingdon, Oxfordshire, United Kingdom

**Keywords:** PRAME, TCR bispecific, melanoma, immunotherapy

## Abstract

**Background:**

PRAME (*Pr*eferentially expressed *A*ntigen in *Me*lanoma) is a cancer–testis antigen expressed in several tumor indications, representing an attractive anticancer target. However, its intracellular location limits targeting by traditional methods. PRAME peptides are presented on the surface of tumor cells by human leukocyte antigen (HLA) molecules, indicating that a T cell receptor (TCR)-based strategy that redirects T cells to kill PRAME^+^ tumors could be a novel immunotherapeutic option. We confirm that PRAME protein is expressed in cutaneous melanoma, including rare subtypes with limited treatment options, as well as primary and metastatic lung, breast, endometrial, and ovarian tumors. Furthermore, PRAME is expressed homogeneously across tumors with distinct oncogenic mutations, mutation burden, PD-L1 expression, immune infiltration, and features of immune checkpoint resistance. Immunopeptidomic analysis of primary tumors detected HLA class I-restricted PRAME peptides.

**Methods:**

A TCR recognizing PRAME peptide SLLQHLIGL was engineered to high affinity and fused to a CD3 engaging domain to create a TCRxCD3 bispecific molecule (*I*mmune-*m*obilizing *m*onoclonal TCR *A*gainst *C*ancer, ImmTAC®) with the ability to redirect polyclonal T cells to efficiently kill PRAME^+^ cells.

**Rs:**

The degree of T cell activation was positively correlated with peptide–HLA abundance, with as few as 10 epitopes per cell sufficient for target cell killing. Impaired ImmTAC®-redirected cytotoxicity of exhausted T cells was rescued using an anti-PD-1 antibody, supporting the use of a combination strategy to treat tumors with active PDL1-PD1 axes.

**Conclusions:**

Our data demonstrate selective and efficient T cell activation and killing by a PRAME-directed TCRxCD3 bispecific, supporting further investigation in multiple cancer indications.

## Graphical Abstract

**Graphical Abstract:**
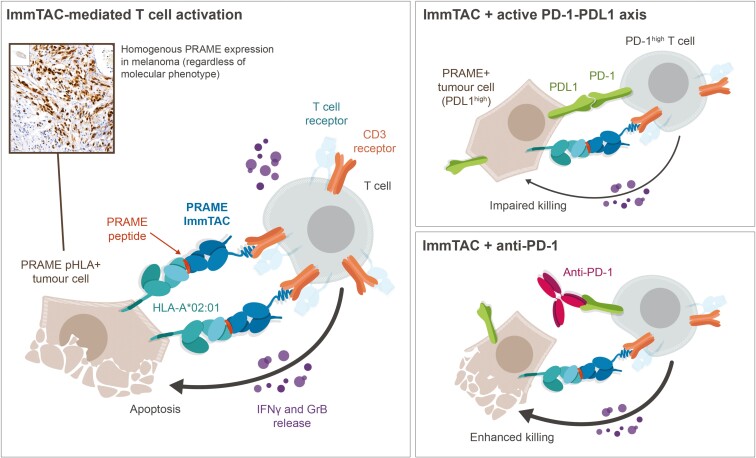

## Introduction

The identification of tumor-associated antigens that are recognized by T lymphocytes to induce tumor-directed immune responses has been a significant area of oncology research in recent years. One group of such antigens are cancer–testis antigens (CTAs) because of their restricted expression profile in cancer, germline, and reproductive organs (e.g. testis, ovary, endometrium, and placenta) [1]. The PRAME (*Pr*eferentially expressed *A*ntigen in *Me*lanoma) protein is an intracellularly expressed CTA initially identified in metastatic cutaneous melanoma [2]. The physiological functions of PRAME are not fully understood, but reports suggest that it serves as a repressor of retinoic acid receptor signaling and inhibits retinoic acid-induced differentiation, growth arrest, and apoptosis [3]. Nevertheless, the prominence of PRAME expression in malignancies and its low levels in healthy tissue render this protein a valuable biomarker for both cancer diagnoses and resection margins during oncology surgery [4, 5]. Indeed, an anti-PRAME monoclonal antibody (EPR 20330) is already being used for the detection of malignant melanoma. PRAME expression is also considered an indicator of poor prognosis in several tumor types, including breast cancer, uveal melanoma, hepatocellular carcinoma, Hodgkin’s lymphoma, and neuroblastoma [6–10]. Moreover, in both primary breast cancer and uveal melanoma, higher PRAME expression levels correlate with an increased rate of distant metastases [6, 8] and may promote epithelial-to-mesenchymal transition during tumor invasion [11].

Because of its association with multiple tumor indications, PRAME is an attractive target for cancer immunotherapy. To date, several strategies evaluating PRAME-specific adoptive T cell therapies and cancer vaccines have been explored [12–14]. PRAME-specific human leukocyte antigen (HLA)-A*02:01-restricted T cell clones have been identified and expanded *ex vivo* [12, 15, 16], and several clinical trials are underway to study the safety and efficacy of this approach [11]. Development of peptide vaccines using recombinant PRAME has so far yielded limited success in advanced solid tumors due to failure to induce an effective CD8^+^ cytotoxic T cell response [17–19]. Another approach for targeting PRAME^+^ tumors is through an emerging new class of T cell-redirecting therapeutics, which rely upon the specificity of the T cell receptor (TCR). Importantly, this strategy provides access to intracellular antigens, presented as peptide–HLA complexes on the cancer cell surface, and exploits a patient’s immune system without the need for manipulation. ImmTAC® (*I*mmune-*m*obilizing *m*onoclonal *T*CR *A*gainst *C*ancer) molecules are bispecific fusion proteins consisting of a soluble affinity-enhanced TCR targeting domain fused to an anti-CD3 single chain fragment variable (scFv) effector domain, which can recruit any available CD3^+^ T cell in an HLA-restricted manner [20]. The first ImmTAC® evaluated in the clinic, Tebentafusp, was shown to have monotherapeutic activity in advanced melanoma and, based on survival benefit in a Phase III randomized clinical trial, has been approved for the treatment of HLA-A*02:01^+^ metastatic uveal melanoma [21–23]. Tebentafusp was shown to increase T cell recruitment into tumors that typically have poor immune infiltration, providing novel benefits where checkpoint inhibitors have demonstrated limited efficacy [24].

Here we show that PRAME is expressed homogenously and at high frequency across multiple tumor indications, in primary and metastatic lesions. We confirmed PRAME peptide presentation by class I HLA molecules and engineered an affinity-enhanced human TCR specific to an HLA-A*02:01-restricted PRAME-derived peptide (SLLQHLIGL). This TCR was reformatted as a bispecific ImmTAC® molecule IMC-F106C, shown to selectively and efficiently redirect T cells to target PRAME^+^ cancer cells. These findings highlight the potential of high-affinity ImmTAC® bispecifics as effective immunotherapies to treat cancers with clear unmet medical needs.

## Materials and methods

### Analysis of PRAME expression in primary tumors by immunohistochemistry

Sections from commercially sourced primary and metastatic tumor samples and tissue microarrays (TMAs) were stained using anti-PRAME antibody (clone EPR20330; ab219650, Abcam and clone E7I1B; 56426S, CST), Hematoxylin counterstain, and DAB (3,3’-Diaminobenzidine). Images were digitized and PRAME protein expression was quantitated based on staining intensity and abundance. Samples with >10% of nuclei with staining were classed as positive.

### PRAME transcript and association analysis

Publicly available *PRAME* mRNA expression data were obtained from The Cancer Genome Atlas (TCGA; http://cancergenome.nih.gov/) and George *et al.* [25] and reanalyzed using QIAGEN OmicSoft Suite. Processed single-cell *PRAME* expression data were obtained from Jerby-Arnon *et al*. [26] and malignant cells were extracted using the corresponding immune resistance annotations. Data were visualized in R (v4.2.2) using Seurat (v4.3.0).

### Analysis of peptide–HLA complexes by liquid chromatography with tandem mass spectrometry

Commercially sourced primary tumor specimens [cutaneous melanoma; ovarian serous adenocarcinoma, non-small cell lung (NSCLC) adenocarcinoma, NSCLC squamous carcinoma (SCC)] were prepared for liquid chromatography with tandem mass spectrometry (LC-MS/MS) (see extended methods section). Briefly, pHLA complexes were captured by affinity chromatography with HLA class I-specific antibodies, eluted, desalted, and analyzed by LC-MS/MS. For absolute quantification and validation, stable isotope-labeled peptides (JPT Technologies, Berlin, Germany) were introduced into each sample. Mass spectrometry (MS) data was processed with PEAKS7.5 (Bioinformatics Solutions) and searched against a human protein database downloaded from Uniprot (uniprot.org).

### In vitro ImmTAC®-mediated T cell activation and killing assays

ImmTAC® molecules targeting PRAME peptide–HLA were engineered as described previously [20, 27, 28]. Tumor cell lines (Table S1; obtained from multiple sources) were used to measure T cell activation by IFNg ELISpot (BD Biosciences) and T cell killing by xCELLigence (RTCA, Agilent) impedance-based cytotoxicity assays. Experiments were performed with healthy peripheral blood mononuclear cell (PBMC) effectors and target cells in the presence of increasing concentrations of ImmTAC® [27]. HLA-A*02:01^+^ patient-derived tumor organoids were expanded as previously described [29]. Organoid coculture assays were performed with healthy PBMC effectors and target cells in the presence of increasing concentrations of ImmTAC®. Tumor cell killing was assessed by high-content imaging of activated Caspase-3/7.

### Generation of PRAME knockout MEL624 and rescue

PRAME knockout (KO) MEL624 cells were generated by Clustered Regularly Interspaced Short Palindromic Repeats (CRISPR) editing using PRAME-specific guide RNA molecules. Edited clones were confirmed by genomic cleavage assays and DNA sequencing. As MEL624 cells have three PRAME alleles, two rounds of CRISPR editing were carried out, generating biallelic (K05) and tri-allelic (A08) KO clones. To restore PRAME expression in MEL624-A08 tri-allelic KO, cells were electroporated with full-length human PRAME IVT RNA (Eurofins) prior to ELISpot and epitope counting with a PRAME ImmTAC® tool molecule that is closely related to IMC-F106C.

### pHLA quantification

Cell surface PRAME pHLA epitope counts were performed by semiautomated microscopy based on Bossi *et al*. [30]. Briefly, cell lines were labeled with fluorescently tagged an IMC-F106C-related ImmTAC®, followed by Annexin V (BioLegend) for live/dead discrimination. Cells were imaged using Ti2 Nikon microscope and the epitope number was measured using NIS® Element 3D spot detector software.

### Tumor-infiltrating lymphocyte isolation

Commercially sourced fresh melanoma biopsies were dissociated to obtain single cell suspension. Tumor-infiltrating lymphocytes (TILs) were enriched using a Pan-T Cell Isolation Kit (Miltenyi, Biotec) and then stained with anti-CD8 APC antibody (BD Biosciences), anti-PD-1 PE antibody, and Zombie Green (both Biolegend). Live CD8+ T cells were Fluorescence-activated cell sorted (FACS) based on PD-1 expression.

### In vitro T cell exhaustion model

PBMCs were isolated from healthy human donor blood and untouched T cells were purified using a Pan-T Cell Isolation Kit (Miltenyi Biotec). *In vitro* T cell exhaustion was induced based on Balkhi *et al*. [31]. In brief, cells were repeatedly stimulated with Human T-Activator CD3/CD28 Dynabeads™ (ThermoFisher Scientific) and IL-2 (Proleukin Novartis) every 3–4 days and assessed by flow cytometry after each stimulation for markers for T cells and T cell exhaustion (CD8, CD4, PD-1, IL7Ra, LAG-3). After four stimulations, T cells were FACS sorted based on PD-1 expression prior to killing assays.

### Statistical analysis

The Wilcoxon test was used to assess *PRAME* expression between sample groups. Tests were two sided and were carried out using R (v4.1). The abundance of tissue-infiltrating immune cell populations was determined using MCP-counter version 1.2 [32]. Transformed PRAME counts per million were generated using edgeR (v3.34) [33]. Euclidean distance was applied per patient to generate a distance matrix and complete-linkage clustering was carried out (R ComplexHeatmap v2.3) [34].

## Results

### PRAME protein is expressed widely in primary tumors of multiple cancer types

**Figure 1.**
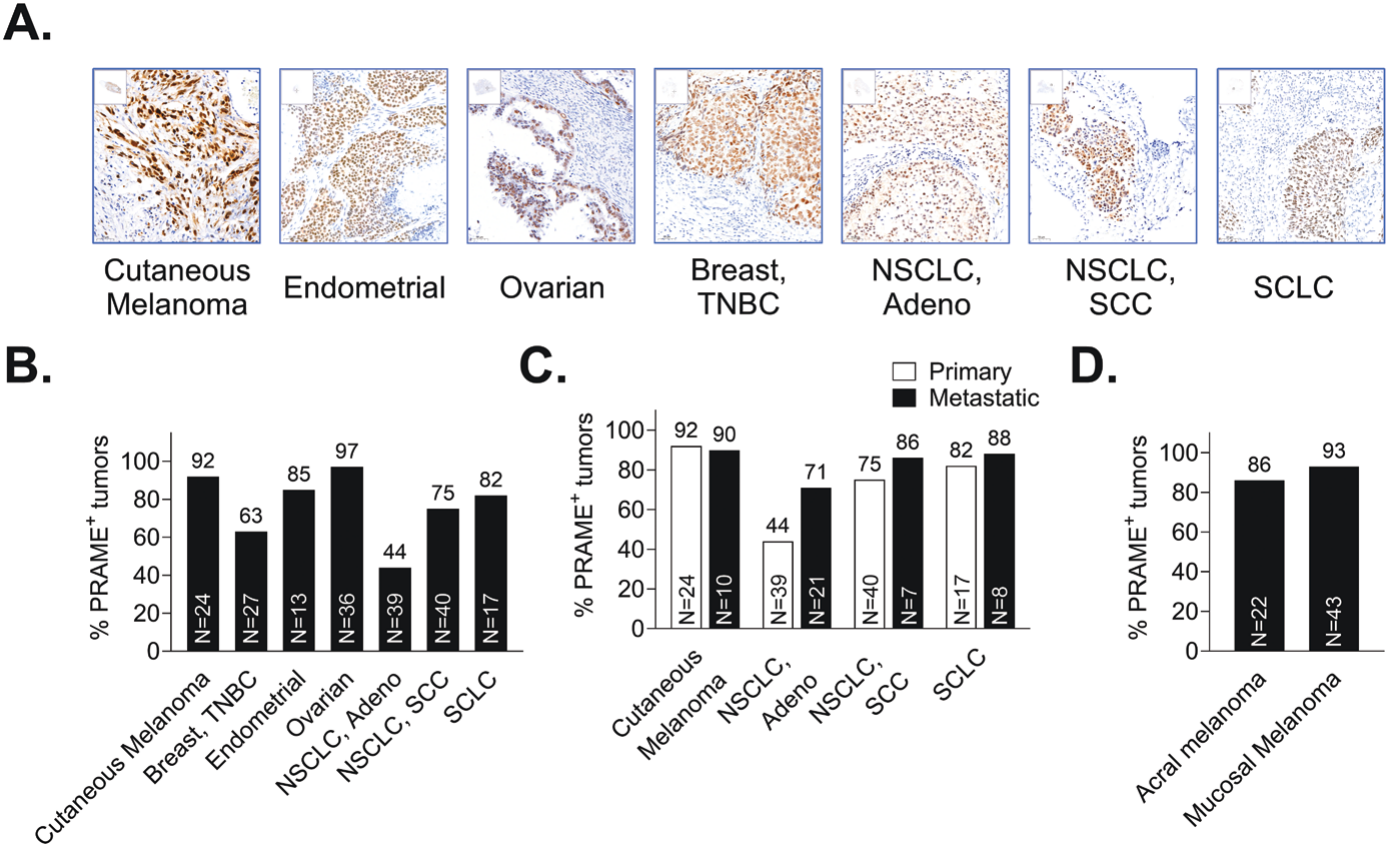
PRAME protein is homogeneously expressed in patient tumors of multiple types and retained in metastasis. (A) Representative IHC staining images of primary tumor types. PRAME prevalence in (B) primary tumor tissues, (C) primary and metastatic melanoma and lung tumor, and (D) acral and mucosal cutaneous melanoma subsets. The number of samples analyzed is indicated for each tumor type.

To confirm the viability of PRAME as a potential target for TCR-directed cancer immunotherapy, protein expression was determined by immunohistochemistry (IHC). PRAME was homogenously expressed across all skin, endometrial, ovarian, breast, and lung cancer types tested (Fig. 1a), suggesting that a PRAME-targeted therapy has the potential to act on most malignant cells within the tumor microenvironment. In line with previous studies [5, 35], PRAME expression was detected in multiple highly prevalent primary tumors, with frequencies >90% in skin cutaneous melanoma (SKCM), and ≥75% in endometrial, ovarian, NSCLC) SCC, and small cell lung cancer (SCLC) (Fig. 1b). In addition, 63% of triple-negative breast cancer (TNBC) and 44% of NSCLC adenocarcinoma (Adeno) tumors showed PRAME staining. PRAME expression was retained in metastatic melanoma and lung tumor lesions (Fig. 1c). PRAME expression was also detected at high frequency in acral and mucosal cutaneous melanoma (Fig. 1d), rare subtypes that are biologically and clinically distinct from cutaneous melanoma, with limited therapeutic options [36]. To confirm the cancer and testis-restricted expression of PRAME, gene expression was analyzed by RNA sequencing in a panel of 40 normal human tissues from the Genotype-Tissue Expression (GTEx) dataset as well as cutaneous melanoma primary tumor from TCGA (Fig. S1a). The average PRAME expression was found to be below 5 FPKM in all tissues with the exception of testis (average expression of 79 FPKM). Additionally, protein expression of PRAME was evaluated by IHC in a panel of 39 normal human tissues from formalin fixed paraffin embedded (FFPE) TMA, resections, and blood smears. Negative protein expression (Fig. S1b) supported the gene expression data.

### *PRAME* transcript is expressed in molecular heterogeneous tumors

**Figure 2.**
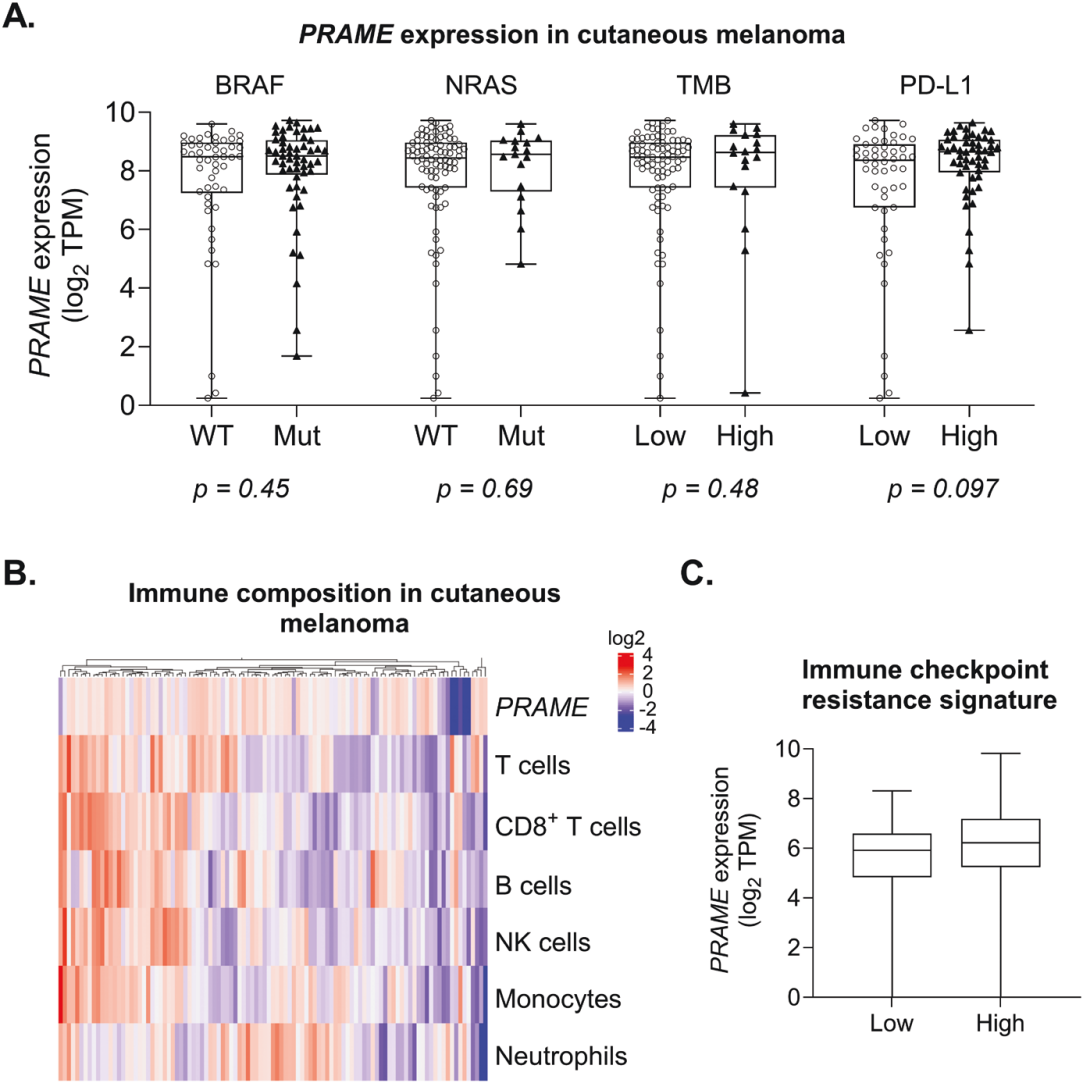
*PRAME* transcript is widely expressed in melanoma independently of oncogenic driver mutations, mutation burden, immune infiltration status, and checkpoint therapy response. (A) Analyses of *PRAME* expression in individual melanoma patient tumors within the depicted groups (BRAF: serine/threonine protein kinase B-Raf; NRAS: GTPase N-Ras; TMB: tumor mutational burden; PD-L1). The Wilcoxon test was used to compare *PRAME* expression between sample groups. (B) Pathway association analyses after unsupervised clustering of bulk melanoma tumor RNAseq, comparing *PRAME* expression with the presence of infiltrating immune populations. (C) scRNAseq data from Jerby-Arnon *et al*. [26] relating low and high immune resistance states to *PRAME* expression across cutaneous melanoma tumors.

One of the hallmarks of cancer is molecular heterogeneity, which is related to genetic and phenotypic variations, observed among individuals with the same tumor type, and can be associated with sensitivity or resistance to targeted therapies. For example, there is a subset of cutaneous melanomas with activating mutations in serine/threonine protein kinase B-Raf (BRAF) or GTPase N-Ras (NRAS), another subset with high tumor mutation burden and sensitivity to checkpoint inhibitors, or another with neither BRAF or NRAS mutations and low tumor mutation burden. To examine whether PRAME-directed TCR-based immunotherapy could be used to treat distinct tumor subsets, we first carried out bioinformatics analysis using TCGA. We analyzed PRAME transcript levels in cutaneous melanoma specimens bearing relevant signaling pathway mutations (Fig. 2) and observed no significant difference in PRAME expression in melanomas harboring mutant BRAF or NRAS genes compared with their wild-type counterparts (Fig. 2a). We extended this analysis to NSCLC adenocarcinoma and SCC subtypes for mutations in epithelial growth factor receptor (EGFR), Kirsten-ras small G-protein oncogene (KRAS), serine/threonine kinase-11 (STK11) tumor suppressor genes, and Kelch-like ECH-associated protein 1 (KEAP1), a redox-sensitive transcriptional modulator (Fig. S1). PRAME expression was overall similar in NSCLC harboring EGFR, KEAP1, KRAS, and STK11 mutations compared with wild-type tumors (Fig. S1a, b).

The ability of tumors to respond to immune checkpoint inhibitors can be associated with their Tumor Mutational Burden (TMB) index, which reflects a total number of mutations [37]. Additionally, programmed death-ligand 1 (*PD-L1*) levels and baseline immune infiltration status can also be associated with sensitivity to checkpoint inhibitors [38]. *PRAME* was broadly expressed across cutaneous melanoma (Fig. 2a) and lung tumors (Fig. S2) with distinct TMB and *PD-L1* biomarker profiles, with some enrichment in NSCLC adenocarcinomas with higher TMB and lower PD-L1 levels (Fig. S2a). This enrichment was not observed for NSCLC SCC (Fig. S2b). To examine the baseline immune landscape in tumors expressing *PRAME*, we performed a correlation analysis of gene expression associated with the main immune cell types in cutaneous melanoma (Fig. 2b) and lung cancer samples using bulk RNAseq data (Fig. S2c). In both melanoma and lung datasets, a proportion of PRAME^+^ tumors have pre-existing immune infiltrates, including T cells. However, many other PRAME^+^ tumors are relatively immune poor. To further understand *PRAME* expression and immune checkpoint blockade resistance, we analyzed *PRAME* expression in 33 melanoma samples previously characterized by Jerby-Arnon *et al.* using single-cell RNA sequencing [26]. *PRAME* is observed at similar levels in tumors with both high and low immune resistance profiles (Fig. 2c). Collectively, these data indicate that *PRAME* is well expressed in a diversity of tumors exhibiting oncogenic mutations or cellular and molecular features associated with sensitivity or resistance to immune checkpoint inhibitors, e.g. tumor mutation burden or immune infiltration.

### PRAME-derived peptide SLLQHLIGL presentation by HLA class I complexes by cancer cell lines and primary tumors

**Figure 3.**
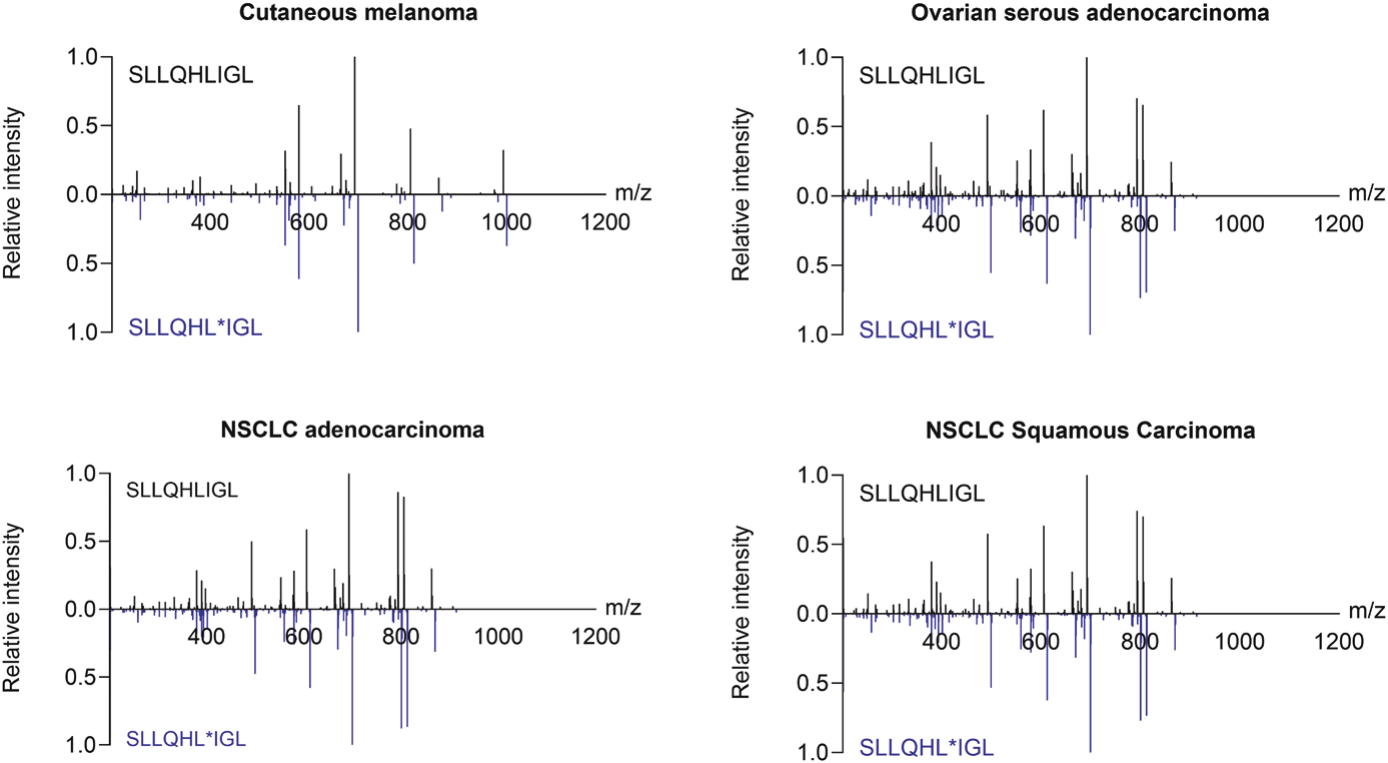
SLLQHLIGL PRAME-derived peptide is presented in the context of HLA class I across different cancer indications. Mirror plots of LC-MS/MS spectra obtained for native peptide (SLLQHLIGL, top) and stable isotope-labeled peptide (SLLQHL*IGL, bottom) in representative examples of cutaneous melanoma, ovarian carcinoma, and NSCLC samples.

To confirm the presentation of PRAME-derived peptides by HLA class I molecules, immunopeptidomic analyses were performed on a large set of HLA-A*02:01^+^ immortalized cell lines (data not shown) and tissue specimens of multiple cancer types. HLA-A*02:01–peptide complexes were immunoprecipitated using an HLA-A*02-specific monoclonal antibody and eluted peptides were analyzed by MS. Figure 3 shows the detection and validation of the most abundant HLA-A*02:01 PRAME-derived peptide SLLQHLIGL in representative samples from multiple indications. Similar retention time and stoichiometries between peptide fragments in both endogenous (top half of graph) and stable isotope-labeled peptide standard (bottom half of graph) confirm the presentation of SLLQHLIGL peptide by HLA class I molecules. This peptide was also detected in tumor indications with PRAME prevalence >40% by IHC (melanoma, ovarian, and lung cancers) (Fig. 1), suggesting a correlation between protein expression and peptide presentation.

### Affinity-matured bispecific ImmTAC® molecules redirect T cells to kill tumor cells expressing PRAME

The results above demonstrated broad PRAME gene and protein expression across diverse tumor cells and detection of the HLA-A*02:01 PRAME-derived peptide SLLQHLIGL in several tumor types. Next, we generated bispecific ImmTAC® molecules that recognize the identified PRAME-pHLA complex; T cells recognizing the cognate PRAME pHLA were isolated from the blood of 26 HLA-A*02:01^+^ healthy donors through target specific *in vitro* stimulation to generate T cell clones (Fig. S3a). Ten unique TCRs were identified through this method, including the leading TCR, which had an affinity of 142 µM [dissociation constant (*K*_D_)] to cognate pHLA assessed by Biacore™ as well as no cross-reactivity to a panel of irrelevant pHLA (data not shown). In order to generate a TCR with sufficient affinity to be potent as a bispecific T cell engager, the TCR was affinity matured through a classical phage display approach using NNK mutagenesis (see supplementary methods). First-generation affinity maturation libraries were constructed targeting each of the six CDRs. Second-generation libraries were built on two templates, a CDR3α mutant (*K*_D_ 3.2 µM, *t*_1/2_ 35.5 s) and a CDR3β mutant (*K*_D_ 12.3 µM, *t*_1/2_ 1.5 s). Affinity-enhancing variants were identified in all loops except CDR1β and combined to generate a panel of TCRs spanning 5 orders of magnitude in affinity (Fig. S3b). A variant was selected (*K*_D_ 2.34 nM, *t*_1/2_ 29 min) for a final round of phage display targeting CDR3α to reach picomolar affinity (see Fig. S3b). Selected picomolar affinity TCRs were fused to a humanized variant of UCHT1 anti-CD3 scFv engaging domain, which mediates activation of T cells to kill cells presenting the target peptide–HLA complex [27]. Using this strategy, multiple PRAME ImmTAC® molecules were screened for potency and specificity and a lead candidate (IMC-F106C) was selected (*K*_D_ <55 pM and binding *t*_1/2_ >12 hours). This lead candidate was used to investigate PRAME-mediated T cell redirection toward cytotoxicity by monitoring IFNγ secretion and direct cytolysis.

**Figure 4.**
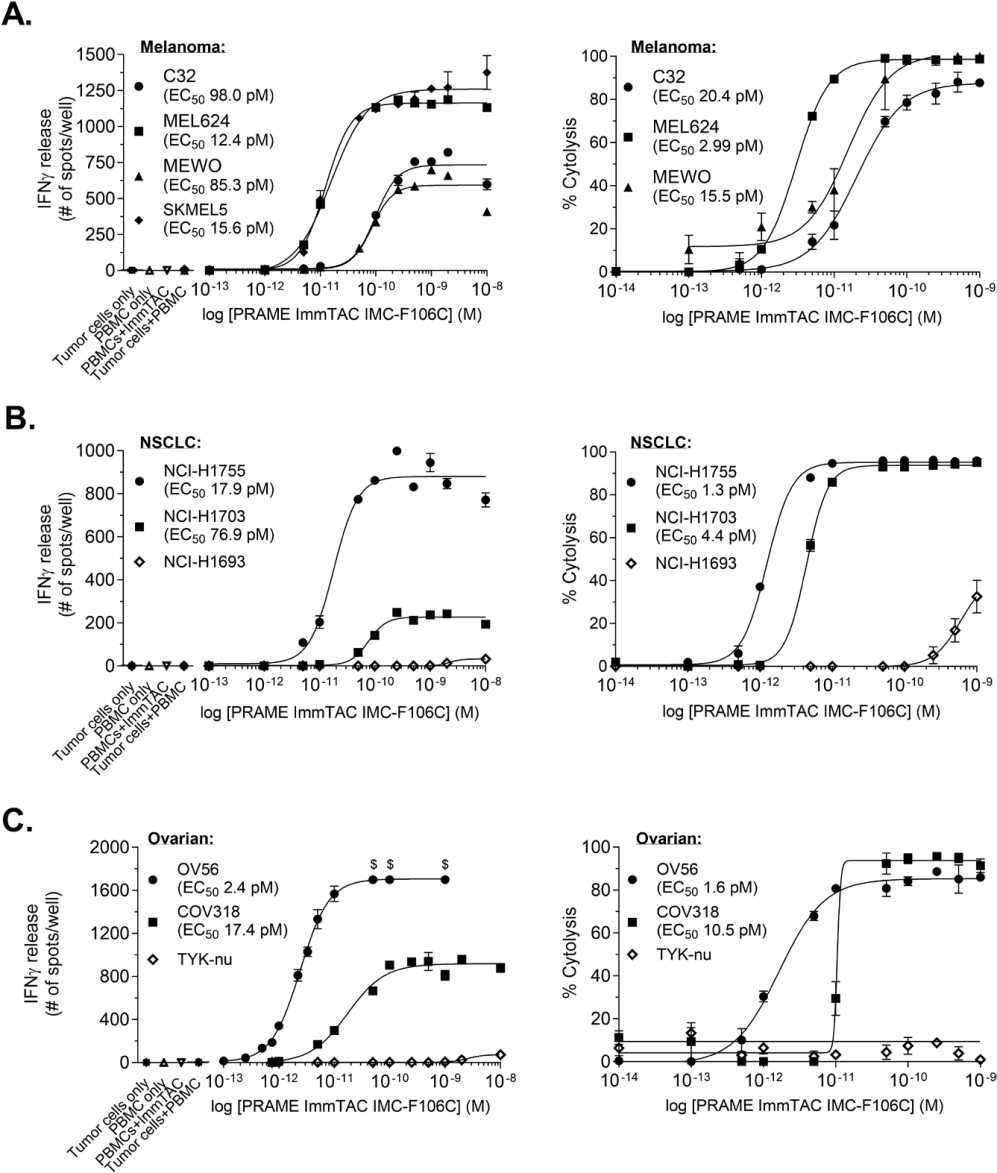
An affinity-enhanced bispecific CD3/TCR ImmTAC® molecule mediates T cell activation and redirected killing of melanoma, lung, and ovarian tumor cells expressing PRAME. HLA-relevant PRAME^+^ melanoma (A), NSCLC (B), and ovarian (C) tumor cells were incubated with PBMC effector cells in the presence of increasing concentrations of the PRAME ImmTAC® IMC-F106C. Ovarian TYK-nu (PRAME^–^/HLA-A*02:01^+^) and lung NCI-H1693 (PRAME^+^/HLA-irrelevant) cell lines were included as controls. Effector cells were also cultured with 2 nM IMC-F106C in the absence of target cells, as control. ImmTAC®-mediated T cell activation was determined by IFNγ ELISpot (left panels). $ represents spots too numerous to count by the software and attributed the highest counted value. IMC-F106C-mediated T cell killing was determined as % tumor cell cytolysis in xCELLigence impedance-based assays (right panels). Data were obtained using three healthy PBMC donors and representative data are shown.

IMC-F106C efficiently activated T cells toward melanoma (MEL624, MEWO, SKMEL5, and C32—Fig. 4a), NSCLC (NCI-H1755 and NCI-H1703—Fig. 4b), and ovarian (OV56 and COV318—Fig. 4c) cancer cells that express PRAME target (i.e. PRAME^+^/HLA-A*02:01^+^) in a dose-dependent manner (representative PBMC donor is shown; additional PBMC donor data can be found in Fig. S4). For all PRAME^+^/HLA-A*02:01^+^ cell lines tested, we observed EC_50_ values for T cell activation, measured by IFNγ release, below <100 pM, even if maximum levels of response varied between cell lines (left panels). In line with this, redirected T cell killing was observed for all PRAME^+^/HLA-A*02:01^+^ cell lines with EC_50_ values <21 pM. In contrast, cancer cells that did not present PRAME-pHLA complexes on their cell surfaces (HLA-A*02:01^–^ NSCLC cells: NCI-H1693 and PRAME^–^ ovarian TYK-nu used as controls for these experiments) did not elicit T cell redirection and activation or cytotoxicity. Importantly, PRAME^-^/HLA-A*02:01^+^ TYK-nu cells demonstrated stimulatory capacity as IMC-F106C-mediated T cell killing is observed when these cells are loaded with exogenous PRAME-derived peptide SLLQHLIGL (Fig. S4d). In addition, IMC-F106C was tested against a wide range of primary normal cells from 10 tissue types and 29 different cell types, including cells of skin and lung origin, to further confirm specificity against PRAME^+^ tumor cells (Fig. S5). No significant reactivity above background (without IMC-F106C) was observed against normal bronchial epithelial cells below 10 nM IMC-F106C and against melanocytes below 1 nM. In contrast, high levels of IFNγ release can be observed for the control PRAME^+^ cell line NCI-H1755 with 0.1 nM of IMC-F106C. Together, these results provide evidence of the potency and specificity of PRAME ImmTAC® IMC-F106C.

To further investigate the ability of IMC-F106C to redirect T cells to selectively kill PRAME^+^/HLA-A*02:01^+^ tumor cells, we developed an organoid model from either PRAME^+^ or PRAME^–^ tumors. Organoids were incubated with T cells and IMC-F106C (10-1000 pM) and caspase activity was assayed as a measure of apoptosis. Results showed Caspase-3/7 activity in PRAME^+^ organoids increased in a dose-dependent manner but remained low in PRAME^–^ organoids (Fig. S6). Collectively, these data indicate that cytotoxic T cells can be effectively redirected by ImmTAC® IMC-F106C to selectively kill PRAME^+^ tumor cells.

### ImmTAC®-mediated T cell redirection is dependent on PRAME antigen levels

**Figure 5.**
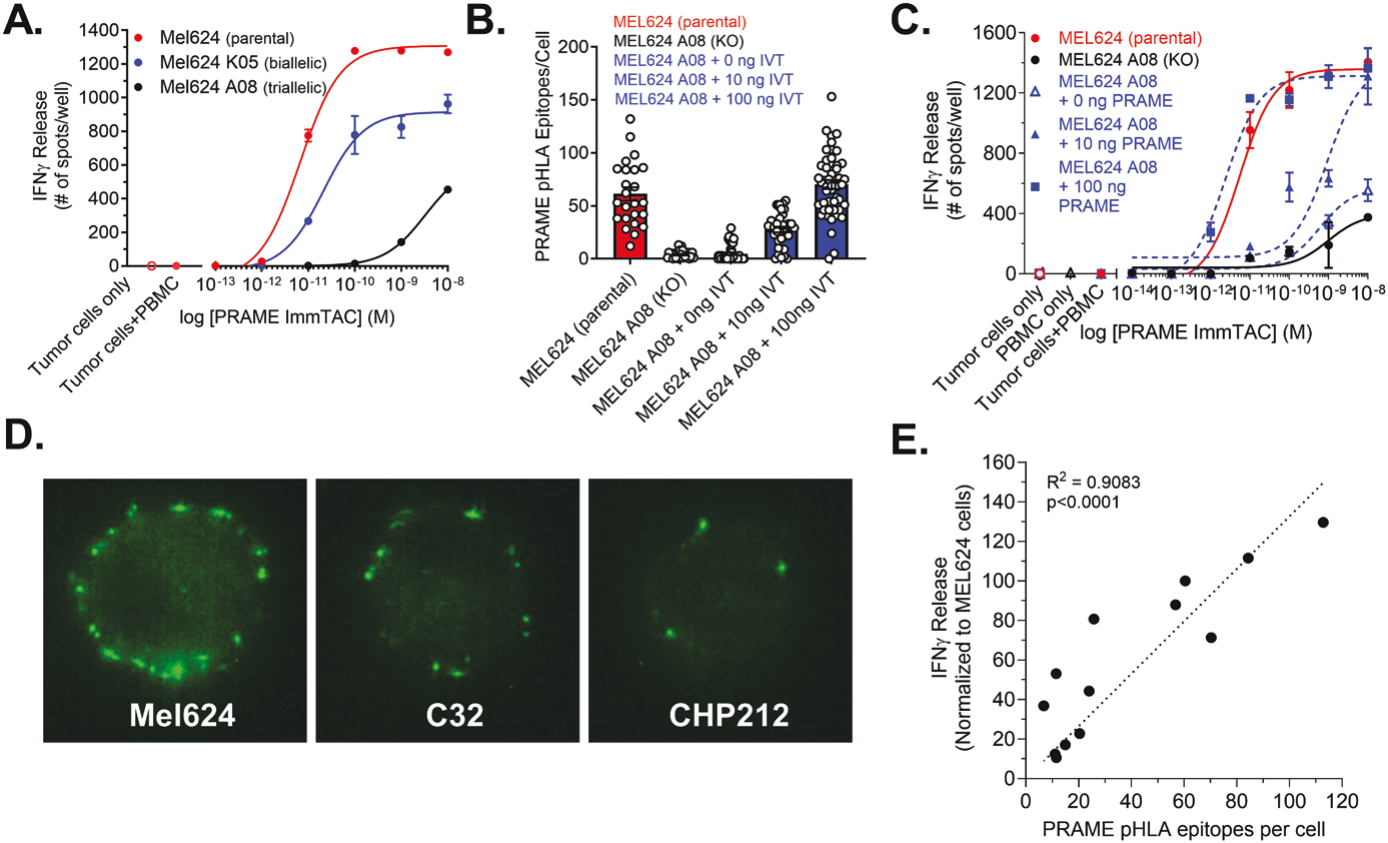
A dose-dependent requirement for intact PRAME antigen provides evidence for the specificity of ImmTAC®-mediated T cell redirection. (A) IFNγ ELISpot analysis of biallelic and tri-allelic deleted MEL624 cell clones K05 and A08 versus wild-type (parental). (B) Representative microscopy data quantitating PRAME-pHLA epitopes for the tri-allelic deleted A08 clone with or without transfection of titrated *PRAME* mRNA sequence (IVT). (C) Cell lines used in (B) were tested in IFNγ ELISpot to demonstrate rescued ImmTAC®-mediated T cell activation. (D) Example fluorescence microscopy images of PRAME-pHLA epitope detection on three cancer cell lines with varying antigen levels; (E) comparison of PRAME pHLA epitope number with the degree of ImmTAC®-dependent T cell activation (IFNγ ELISpot), area under the curve (AUC), relative to MEL624 (set to 100). Data are representative of at least two experiments per cell line. ELISpot assays were performed with cells from the same healthy PBMC donor.

To further confirm the specificity of the PRAME-targeting ImmTAC® molecule, we deleted the target peptide sequence (SLLQHLIGL) in a cutaneous melanoma cell line that expresses abundant PRAME peptide in the context of HLA-A*02:01 (MEL624). As MEL624 cells express up to three separate PRAME alleles, two cycles of CRISPR/Cas 9 gene editing were required to achieve full-scale deletion, resulting in a biallelic (K05) and tri-allelic (A08) clone (Table S2). In contrast to the parental MEL624 cell line, clone A08 was poorly responsive to ImmTAC-redirected T cells, whereas clone K05 showed intermediate reactivity (Fig. 5a). Next, clone A08 was transfected with increasing concentrations of full-length PRAME expression vector, and restored expression of PRAME-pHLA complexes was confirmed by fluorescence microscopy (Fig. 5b). When these engineered cells were challenged with T cells and a PRAME-targeting ImmTAC, there was a dose-dependent increase in IFNγ secretion, indicative of T cell activation (Fig. 5c). These data confirm that the intact PRAME-SLLQHLIGL peptide is required for specific ImmTAC®-mediated T cell redirection and activation *in vitro*.

### ImmTAC®-dependent redirection of T cells requires as few as 10 surface PRAME-pHLA complexes

Widespread expression of PRAME in primary tumors and metastatic lesions underpins the interest in using it as a target for T cell redirection. However, there is a range of *PRAME* expression among tumor cell lines and primary tumor specimens that can affect the level of ImmTAC®-mediated responses (Figs 4 and 5c). To explore the potential of the PRAME-specific ImmTAC® to redirect T cells in cancer cells presenting a small number of PRAME-pHLA complexes, cell lines with varying levels of PRAME or HLA-A were probed with fluorescence-labeled PRAME-targeting ImmTAC®. PRAME-pHLA complexes on the surface of viable cells were quantified using confocal microscopy. These experiments confirmed variable levels of PRAME-pHLA complexes between cell lines (Fig. 5d); melanoma MEL624 cells, which have high PRAME expression genotype, had abundant complexes on the plasma membrane, whereas glioblastoma CHP212 cells did not. C32 melanoma cells show intermediate levels of PRAME-pHLA complexes.

We then evaluated the relationship between the relative number of PRAME-pHLA complexes on the plasma membrane with their ability to activate T cells in an ImmTAC®-dependent manner (see Table S1 for details on tumor cell lines). There was a positive linear correlation between cell surface PRAME-pHLA complexes and T cell activation, measured by IFNγ release (Fig. 5e). T cell activation was detected against target cell populations with as few as ~10 pHLA epitopes. All cell lines shown to elicit T cell-mediated responses were also sensitive to T cell killing in the presence of 100 pM ImmTAC® at 24 hours (data not shown). Collectively, our data demonstrate that the PRAME-targeting ImmTAC® can redirect T cells to selectively and efficiently kill PRAME^+^/HLA-A*02:01^+^ tumor cells with varying levels of PRAME expression and peptide presentation.

### PRAME-targeting ImmTAC® activity is rescued by combination with checkpoint inhibitors in melanoma tumors with an active PD-1/PD-L1 axis

**Figure 6.**
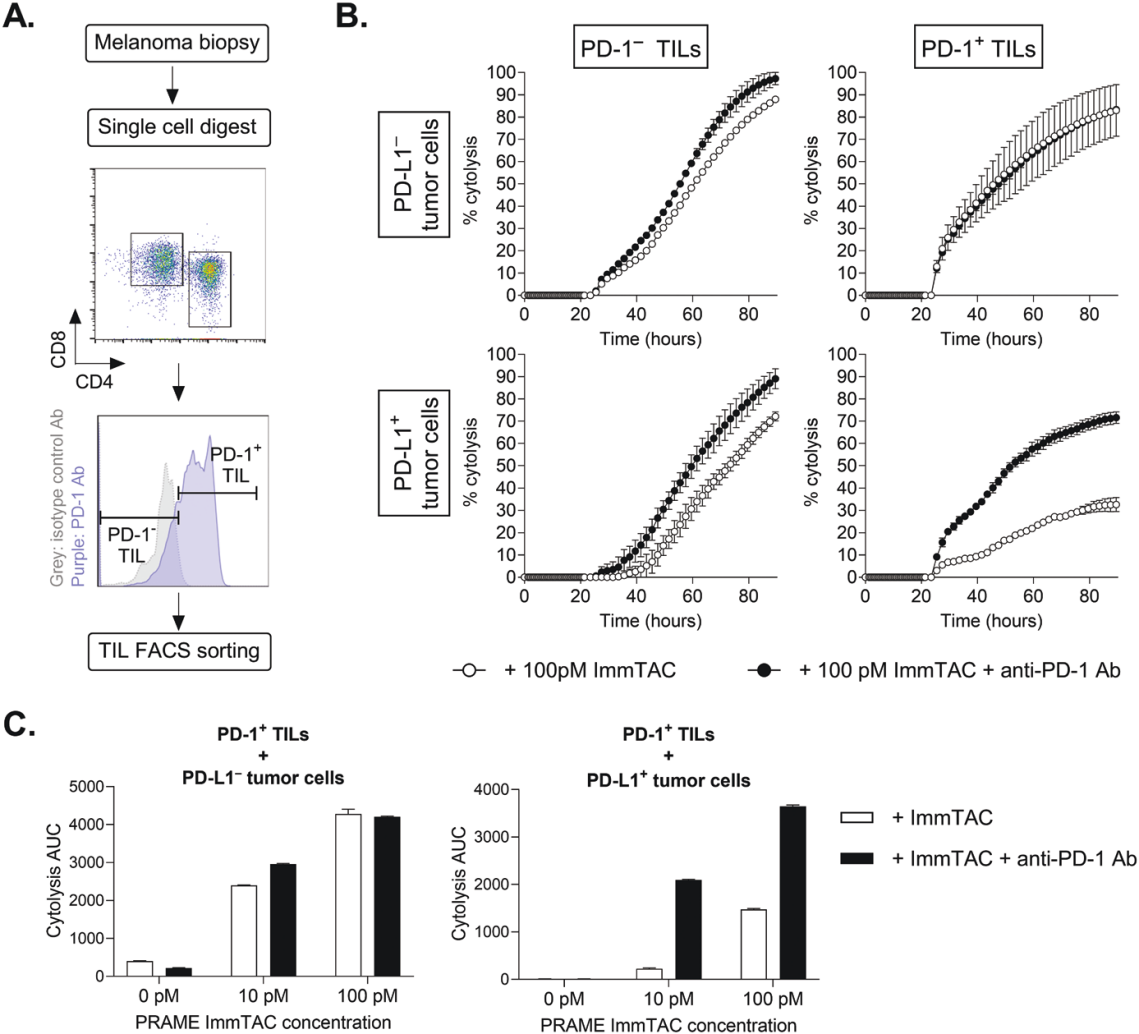
PRAME-targeting ImmTAC® redirects PD-1^+^ TILs to kill tumor cells, an effect that is reduced against PD-L1-expressing tumor cells, but alleviated by anti-PD-1 Ab. (A) CD8^+^ TILs from a melanoma tumor sample were FACS sorted into programmed cell death protein 1 (PD-1)^+^ and PD-1^–^ populations. (B) ImmTAC®-mediated redirection of PD-1^–^TILs (left panels) and PD-1^+^ TILs (right panels) resulted in the killing of PD-L1^-^ tumor cells (MEL624, top panels) and PD-L1^+^ tumor cells (PD-L1 transduced MEL624 cells, bottom panel). The experiment was performed over 4 days in the absence (white circles) or presence (black circles) of blocking anti-PD-1 antibody. (C) % cytolysis area under the curve (AUC) was calculated for the conditions presented in (B), using increasing ImmTAC® concentrations in the presence (black) or absence (white) of anti-PD-1 antibody. Data are shown as mean ± SEM.

Persistent T cell stimulation within the tumor microenvironment induces high PD-1 expression, leading to a state of T cell exhaustion whereby T cells gradually lose effector functions. The ability of the PRAME-targeting ImmTAC^®^ to redirect PD-1^+^ and PD-1^–^ T cells was assessed *ex vivo*. PD-1^+^ and PD-1^–^TILs were isolated from melanoma tumor biopsies (Fig. 6a) and redirected by ImmTAC to kill tumor cells that were either PD-L1^+^ or PD-L1^–^. Purified PD-1^+^ (exhausted) TILs were as efficient as PD-1^–^ TILs in killing PD-L1^–^ tumor cells in the presence of 100 pM of ImmTAC®, and the addition of anti-PD1 blocking antibody had no impact (Fig. 6b and c). However, when co-incubated with PD-L1^+^ tumor cells, PD-1^+^ TILs showed a 3–10-fold reduction in ImmTAC®-mediated killing, which was reversed by the addition of anti-PD1 antibody (Fig. 6b and c).

In parallel, we explored an *in vitro* exhaustion model whereby PD-1 expression was induced in T cells through repetitive stimulation (Fig. S7), based on the Balkhi *et al*. methodology [31]. Repeated stimulation of T cells resulted in gradual loss of IL-7Rα and upregulation of the exhaustion markers PD-1 and LAG3, consistent with an exhausted T cell phenotype (Fig. S7a). Consistent with the results obtained with TILs, ImmTAC^®^ molecules were capable of redirecting exhausted T cells against PD-L1^−^ tumor cells; however, this redirection and killing were reduced up to five-fold against PD-L1^+^ tumor cells (Fig. S7b—left panel). As before, co-incubation with an anti-PD-1 antibody restored cytotoxicity up to nine-fold in these conditions (Fig. S7b—gray bars on the left panel). Together, these results suggest that anti-PD-1 blockade therapy may enhance PRAME-targeting ImmTAC® activity in melanomas or other tumors (e.g. NSCLC) with an active PD-1/PD-L1 axis.

## Discussion

The prevalence of PRAME in diverse tumor types and restricted expression patterns to cancer and testis and ovarian germline cells make it an attractive target for T cell-based immunotherapies [2, 35]. In this report, we performed a systematic analysis of PRAME transcripts, protein, and pHLA expression across multiple solid tumor types, including primary and metastatic malignancies, using in-house specimens and publicly available expression datasets. We demonstrated wide-ranging expression of PRAME mRNA and protein across melanoma subtypes, gynecologic cancers (ovarian and endometrial), lung (SCLC and NSCLC), and TNBC, consistent with previous reports [5, 35]. In these tumors and distant metastases, PRAME shows homogeneous expression, with no expression in adjacent parenchyma or surrounding stroma.

While several tumor types expressing PRAME have established standard-of-care treatments, including checkpoint inhibition in melanoma [39], chemotherapy, and targeted therapy (such as EGFR pathway inhibition and beyond) in lung cancer [40], clinical benefit from such therapies can be transient, with tumors evolving drug resistance, resulting in continued unmet need. For example, EGFR inhibition can select for drug-resistant EGFR mutant tumor cells [41] or some cancers are resistant to immune checkpoint immunotherapies [42]. PRAME was broadly expressed across tumors harboring oncogenic mutations, e.g. BRAF/NRAS-mutated melanoma or KRAS or EGFR mutant NSCLC, in accordance with a previous report [43]. Additionally, PRAME expression was similar in subsets of tumors with molecular features of sensitivity (e.g. TMB or PD-L1) or resistance (e.g. STK11 mutations) to checkpoint inhibitors. These data suggest that PRAME-targeted therapies could have broad antitumor activity.

Initial therapeutic strategies have focused on the selective elimination of PRAME-expressing tumor cells in a variety of cancers, including the development of PRAME protein and peptide vaccines [11]. However, this approach has yielded limited success in NSCLC, as patients failed to mount an effective CD8^+^ T cell response, despite exhibiting humoral responses that stimulate CD4^+^ T cell activation [17–19]. For example, Pujol *et al*. [17] used a proprietary recombinant PRAME molecule as an immunostimulant in a Phase I safety study in NSCLC patients who had undergone surgical resection; anti-PRAME antibodies, but no CD8^+^ responses, were detected following four immunizations. In a separate study, patients with circulating PRAME-specific T cells prior to immunization were less likely to undergo postvaccination T cell expansion and did not exhibit antitumor activity, suggesting these cells may be anergic [18]. Furthermore, PRAME is a self-antigen, and therefore, it is likely that PRAME auto-reactive T lymphocyte clones are rare as they are either deleted during development and maturation in the thymus, or display low affinity against PRAME targets [44]. These caveats suggest alternative approaches that generate specific and durable T cell therapeutics are needed.

Previous studies identified PRAME peptides that are presented on the cell surface of tumors in the context of various HLA genotypes and elicited PRAME-specific CD8^+^ T cells, confirming the immunogenic nature of PRAME-derived peptides [2, 45]. Based on high representation in our MS dataset, the previously described HLA-A*02:01-restricted SLLQHLIGL peptide was selected as a target for the ImmTAC® platform and the studies reported herein [46]. We have further validated PRAME-SLLQHLIGL in primary tumor samples of multiple origins. To isolate PRAME-specific TCRs, blood from a total of 26 healthy HLA-A*02:01^+^ donors was screened using sequential stimulation of T cell minicultures with cognate peptide-pulsed autologous antigen-presenting cells. Low affinity expected for PRAME-specific TCRs was confirmed, with *K*_D_ values between 42 and 500 µM (Fig. S3). Using our ImmTAC® technology, we engineered a bispecific molecule (IMC-F106C) with a high-affinity TCR against the SLLQHLIGL pHLA complex and an anti-CD3 engager. We confirmed that IMC-F106C recognized PRAME-pHLA on the cancer cell surface, redirected donor T cells, and efficiently mounted an anti-PRAME-specific antitumor response (i.e. CD8^+^ IFNγ release and cytolysis). This was demonstrated in cell lines of multiple indications and in patient-derived organoids with confirmed PRAME expression. IMC-F106C was tested against an extended panel of primary normal cell types, including cells from skin and lung origin (Fig. S3); whole blood and HLA-A*02^-^ lymphoblastoid cell lines (EBV-transformed) failing to redirect T cells against cells that were PRAME^–^ or HLA-A*02:01^–^ (data not shown). In addition, the binding motif and potential off-target risks for the IMC-F106C TCR were investigated using an x-scan technique in which every amino acid in the target peptide sequence (SLLQHLIGL) was substituted by any other 19 different amino acids, further confirming the specificity of the PRAME ImmTAC^®^ IMC-F106C (data not shown). While no reactivity was observed against bronchial epithelial cells below 10 nM IMC-F106C, IFNγ levels above the background were detected when testing melanocytes at lower drug concentrations (down to 1 nM) (Fig. S2). This low reactivity suggests that skin-related side effects, such as rash and pigmentation changes, may present in the clinic. Reactivity against primary normal cells of renal origin was observed (data not shown), in line with previous reports of PRAME expression in proximal epithelial cells [12] and, therefore, renal function is also monitored in the clinic. Moreover, the specificity of PRAME ImmTAC® was further confirmed by CRISPR/Cas9 deletion of the SLLQHLIGL peptide-encoding sequence in cancer cells. In preliminary experiments, viable outgrowing clones could not be generated from CRISPR-mediated full knockout of PRAME in MEL624 cells (data not shown), consistent with literature that PRAME inhibition causes cell cycle arrest and apoptosis in leukemic and hepatocellular carcinoma cells [10, 47].

We subsequently quantified the number of peptide–HLA complexes presented on the surface of single cells of PRAME^+^ cancer cell lines using fluorescence microscopy. The number of epitopes varied for the different cell lines analyzed, consistent with tumor heterogeneity between indications and patients. Interestingly, we identified a strong correlation between the number of epitopes presented by target cells and their ability to elicit T cell responses in the presence of PRAME ImmTAC®. To our knowledge, this is the first time that immunogenic PRAME peptide quantification in live cells has been reported. Furthermore, we show the affinity-enhanced PRAME ImmTAC is able to elicit an antitumor response with as few as 10 epitopes per cell, suggesting efficacy in targeting tumor cells with a wide range of PRAME peptide presentation levels. This indicates that ImmTAC®-redirected T cell activation mirrors the high sensitivity of natural TCR-mediated engagement previously reported, with a single TCR-pMHC interaction sufficient to activate helper T cells [48] and three pMHC complexes able to promote cytotoxic T cell killing [49].

A critical factor for oncology immunotherapy is the robust infiltration of cytotoxic immune cells into the tumor microenvironment, permitting the efficient killing of cancer cells. The first approved ImmTAC® molecule, Tebentafusp, has shown therapeutic benefit for uveal melanoma [24], despite relatively poor immune cell infiltration in this tumor type [50]. Indeed, Tebentafusp induces a significant increase in the frequency of CD3^+^ T cells in tumors upon treatment [21]. In this report, we show that *PRAME* is equally expressed in melanoma and lung tumors, independent of immune cell infiltration (Fig. 2b and Fig. S1c). Additionally, we demonstrate that *PRAME* is expressed in cutaneous melanoma specimens that are refractory to immune checkpoint blockade (Fig. 2c). Resistance to checkpoint inhibitor therapy is often associated with low TMB, poor homing of immune cells into the tumor [38, 42], and *PD-L1* expression levels. Importantly, our analyses revealed that *PRAME* was expressed irrespective of overall TMB or *PD-L1* expression levels. Based on Tebentafusp-induced T cell recruitment to tumors, we hypothesize that PRAME-targeting molecules, such as IMC-F106C ImmTAC®, offer a novel means to address PRAME^+^ solid tumors that are resistant to the current standard of care. In addition, this report suggests a potential strategy to address tumors in which the PD-1/PD-L1 axis is active, by combining IMC-F106C with standard-of-care anti-PD-1 blocking antibodies. Although exhausted PD-1^+^ effector T cells are less proficient at ImmTAC®-mediated killing of PD-L1^+^ tumor cells *in vitro*, cytotoxic activity can be rescued with an anti-PD-1 blocking antibody. This combination approach could harness both the power of immune checkpoint blockade and the ImmTAC®-mediated recruitment of T cells from the periphery to enhance the killing of PDL1^+^ tumors.

In summary, our data have confirmed broad expression of *PRAME* across a variety of solid tumor types, including cutaneous melanoma, endometrial, ovarian, lung, and breast cancers. We have demonstrated highly efficient killing of PRAME^+^ tumor cells using our soluble TCR bispecific ImmTAC® molecule (IMC-F106C), even with as few as 10 PRAME-pHLA complexes present. Thus, the novel PRAME-targeting IMC-F106C ImmTAC® represents a highly specific and sensitive immunotherapeutic modality, applicable across a wide range of solid tumors, two critical features for efficient T cell-based cancer therapy. Furthermore, the antitumor activity of IMC-F106C can be enhanced by combining with anti-PD1 agents in tumors with an active PD-1/PD-L1 axis. The safety and antitumor activity of IMC-F106C as monotherapy and in combination is currently being investigated in clinical trials in melanoma and other tumors (NCT04262466, NCT06112314).

## Supplementary Material

ltae008_suppl_Supplementary_Materials

## Data Availability

Data analyzed were obtained from The Cancer Genome Atlas (TCGA, http://cancergenome.nih.gov/); the European Genome-phenome Archive under the accession code EGAS00001000925; the QIAGEN OmicSoft Oncoland TCGA_B38 20190215_v8; and Gene Expression Omnibus (GEO) under the accession number GSE115978, with annotations provided in https://singlecell.broadinstitute.org/single_cell, accession SCP109. The data generated in this study are available upon request from the corresponding author.
