# Supplementary material for: High-affinity T cell receptor ImmTAC® bispecific efficiently redirects T cells to kill tumor cells expressing the cancer–testis antigen PRAME
Source: Immunother Adv. 2024 Nov 2;4(1):ltae008. doi: 10.1093/immadv/ltae008 (PMC11631188; doi:10.1093/immadv/ltae008)
Supplement: ltae008_suppl_Supplementary_Materials [file ltae008_suppl_supplementary_materials.docx]

**EXTENDED METHODS:**

**Analysis of PRAME expression in primary tumors by immunohistochemistry (IHC)**

Primary and metastatic tumor tissues from cancer patients and tissue microarrays (TMA) were formalin fixed paraffin embedded (FFPE) and staining of tissue sections was performed on a Labvision Autostainer (480S, Thermofisher). Quality of preservation and tumor histological features were reviewed for each sample by a qualified pathologist. Isotype control staining confirmed specificity (data not shown). Images were digitized with a whole-slide scanner (P250 Flash, 3Dhistech) and PRAME protein expression was quantitated by a qualified pathologist based on staining intensity and abundance. Samples were classed as positive if >10% of nuclei showed staining at any intensity.

**PRAME transcript and association analysis**

Expression and mutational data from primary melanoma and non-small lung cancer (NSCLC) samples were extracted from QIAGEN OmicSoft Oncoland TCGA_B38 20190215_v8. PD-L1 (CD274) groups are based on median transcript per million (TPM) threshold 1.14 for cutaneous melanoma (SKCM) (3.82 and 4.39 for lung adenocarcinoma (LUAD) and squamous cancer (LUSC), respectively.

**Analysis of peptide-HLA complexes by LC-MS/MS**

Commercially sourced (Proteogenex (USA) and Tissue Solutions (UK)) flash-frozen surgically resected primary tumor specimens (500-1,500 mg gross weight) were stored at -80°C before milling under cryogenic conditions. Pulverized tumor material was lysed and centrifuged. HLA class I peptide complexes were captured by affinity chromatography with anti-HLA-A*02 BB7.2 antibody followed by anti-HLA-class I W6/32 antibody immobilized on a proteinA-Agarose scaffold. HLA-peptide complexes were eluted in acidic conditions and desalted by reversed phase solid phase extraction (Sep-Pak C18, Waters) for LC-MS/MS analysis. Samples were loaded onto an Acclaim™ PepMap™ 100 trap column (100 µm x 2 cm, ThermoFisher); separated using an EASY-Spray™ column (75 µm x 50 cm, ThermoFisher) with mobile phase A (0.1% formic acid in water), and B (0.1% formic acid in ACN) at a flowrate of 250 nL/min; eluted into an EASY-Spray™ ionization source (ThermoFisher) and analyzed on an Orbitrap Fusion™ or Orbitrap Fusion™ Lumos™ Tribrid™ Mass Spectrometer (ThermoFisher). Stable-isotope labeled peptides (JPT technologies, Berlin, Germany) were introduced into each sample at 100 femtomole immediately prior to LC-MS/MS analysis. MS data searched against a human protein database downloaded from Uniprot (uniprot.org) with no enzyme restriction and a tolerance of 5 ppm for MS1 and 0.02 Da for MS2. Carbamidomethylation of cysteine residues was set as fixed post-translational modification. Acetylation, carbamylation, deamidation, oxidation, deoxidation, phosphorylation, pyro-glu from glutamic acid and glutamine were set as variable modifications. Data were exported at 5% estimated false discovery rate (FDR). Parallel reaction monitoring (PRM) data was analyzed using Freestyle (ThemoFisher).

**T cell receptor isolation, affinity maturation and protein production**

TCR isolation and engineering to produce ImmTAC**®** reagents has been described previously (Li et al., 2005; Liddy et al., 2012; McCormack et al., 2013). Briefly, T cells were isolated from blood donated by healthy adult volunteers under a Research Ethics Committee approved UK Health Research Authority study (reference 13/SC/0226). All donors were HLA-A*02:01 positive. T cells were stimulated *in vitro* with autologous antigen presenting cells pulsed with the PRAME SLL peptide: dendritic cells followed by activated B cells. T cell cultures were screened for specificity by IFNg ELISpot against T2 target cells pulsed with SLL peptide vs. an irrelevant control peptide. Positive T cell cultures were re-stimulated with SLL peptide-pulsed T2s and CD8+CD137+ cells were sorted by flow cytometry onto feeder cells (irradiated allogenic PBMC) to generate T cell clones. Following expansion, T cell clone specificity was re-confirmed in IFNg ELISpot. Cells were then lysed and frozen in Trizol reagent. TCR sequences were identified by rapid amplification of cDNA ends (RACE). Engineering the TCR for high affinity was achieved using phage display. Affinity maturation phage libraries were built using overlapping NNK oligonucleotides targeting each CDR segment of TCRα and TCRβ genes. To enrich for the highest affinity clones, successive rounds of panning were performed reducing the amount of target pHLA at each round. Phage competition ELISA was used to screen for affinity-enhanced mutants. To produce soluble disulfide-linked mTCRs and ImmTAC® molecules, TCRα and TCRβ chain inclusion bodies were denatured using 50mM Tris buffer pH 8.1 containing 100mM Sodium Chloride, 6M Guanidine and 20mM Dithiothreitol (DTT) and refolded by diluting to final protein concentration of 60mg/L into a buffer containing 100mM Tris pH 8.1, 4M Urea, 400 mM L-Arginine, 1.9mM Cystamine and 6.5mM Cysteamine redox couple. Refolding mixture was dialysed against water followed by 20mM Tris buffer pH 8.1. Correctly folded protein was purified by anion exchange chromatography, cation exchange chromatography and size exclusion as previously described.

***In vitro* ImmTAC®-mediated T cell activation and killing assays**

Tumor cell lines (Table S1) and primary normal cells were subjected to regular verification of identity by short tandem repeat (STR) profiling. Primary normal melanocytes and bronchial epithelial cells were handled according to supplier’s recommendations. ELISpot assays were performed using 1:1 ratio of HLA-A*02:01^+^ healthy PBMC effectors (E) to target (T) cells and interferon γ (IFNγ) was measured after 24 hours. xCELLigence impedance-based cytotoxicity assays were performed as per manufacturer’s recommendations (ACEA xCELLigence Real-time Cell Analysis, RTCA, Agilent) using 5:1 (E:T) ratio. Tumor cell killing was shown as percent of cytolysis after comparison with signal without drug. For ImmTAC®/anti-PD1 combination studies, T cells or tumor infiltrating lymphocytes (TILs) were added at 2:1 (E:T) ratio in the presence or absence of 100 pM of IMC-F106C-related ImmTAC® and in the presence or absence of 10 µg/ml anti-PD1 antibody (humamised IgG4, Selleckchem). Organoid co-culture assays were performed in triplicate with HLA-matched PBMC from three healthy donors at a 10:1 (E:T) ratio in the presence of ImmTAC®. Tumor cell killing was assessed by high-content imaging using a dye for activated Caspase-3/7, with images masked on organoid-containing regions using brightfield microscopy. Caspase activity at 48 hours was baseline-corrected for the signal in absence of ImmTAC®.

**Generation of PRAME Knock-out (KO) MEL624 and rescue**

The coding sequence of target SLLQHLIGL peptide was deleted from the PRAME^+^ cutaneous melanoma cell line MEL624 by two rounds of CRISPR editing. For the first round of editing, MEL624 cells (three PRAME alleles) were transfected with Cas9-gRNA nucleoprotein complexes (GeneArt Platinum Cas9 nuclease (Invitrogen)) alongside PRAME guide RNA-T1 (produced using primers forward 5ʹ-TAATACG-ACTCACTATAGCTGGCTGTGTCTCCCG-3ʹ and reverse 5ʹ- TTCTAGCTCTAAAACTTGACGGGAGACA-CAGCCA-3ʹ) and PRAME guide RNA-T2 (produced using primers forward 5ʹ-TAATACGACTCACTA-TAGTCAGATTGCTCAGCCC-3ʹ and reverse 5ʹ-TTCTAGCTCTAAAACCATCGGGCTGAGCAATCTG-3ʹ) using Lipofectamine CRISPRMAX and Cas9 Plus reagent (Invitrogen). Resulting monoclones were cultured after limiting dilution. Edited clones were identified by genomic cleavage assay (GeneArt Genomic cleavage detection kit, Life Technologies, and primers 5ʹ-CCCCTTTTTTTCCTCACTGAAC-3ʹ, 5ʹ-CATGCTGACCGATGTAAGTCCC-3ʹ) and subjected to DNA sequencing. One culture (clone K05) exhibited indels within the PRAME-SLLQHLIGL peptide region of two alleles (-2bp yielding SLLQHLMAE and -59+8bp yielding complete deletion of SLLQHLIGL peptide). To disrupt the remaining PRAME allele, MEL624 clone K05 cells were subjected to a second round of CRISPR targeting with Cas9-gRNA-RNP complex (PRAME gRNA CAGATTGCTCAGCCCGATG, Dharmacon) and cultured as above. Edited clones were identified as above and genomic sequencing confirmed subclone A08 had an additionally disrupted third PRAME allele (-13+3bp and +1bp respectively). The tri-allelic targeted clone was authenticated by STR profiling prior to functional analyses. To restore the deficient ImmTAC® response of tri-allelic targeted MEL624-A08, cells were transfected with up to 100 ng full-length human PRAME IVT RNA (Eurofins) per 1x10^5^ cells using Neon electroporation (protocol 22 in buffer E) 4 hours prior to ELISpot assay and epitope counting.

**pHLA quantification**

An IMC-F106C related molecule was fluorescently labelled by addition of CF®640R succinimidyl ester (Biotium) at 10:1 molar ratio for 1 hour followed by a wash with 10k molecular weight cut-off spin filters (Thermofisher). ImmTAC®-dye conjugate concentrations were determined by Lambert-Beer law using A_280_ and A_662_ absorbances. For each staining, cell lines were briefly trypsinized and washed in cell imaging buffer (Invitrogen Cell Imaging Solution) at 4⁰C. Non-specific interactions were blocked by incubation in cell imaging buffer supplemented with 1 mg/mL bovine serum albumin (BSA), 5% goat serum and 100 nM irrelevant ImmTAC® molecule for 1 hour at 4⁰C, followed by labelling with 100 nM fluorescently tagged PRAME ImmTAC® for 1 hour at 4⁰C. Cells were labelled with Annexin V (BioLegend) in accordance with manufacturer’s instructions. Cell pellets were washed and fixed for 15 min with 1x BD™ Stabilizing fixative (BD Biosciences). Cells were washed again and transferred to Poly-L-lysine-coated imaging chambers (Thistle Scientific), then treated with quenching solution (0.1 M Glycine, 0.1 M NH_4_Cl) for 15 min. Cells were washed with PBS and imaged within 24 hours. Imaging was performed using a Ti2 Nikon microscope and NIS® Elements software. Dead Annexin V^+^ cells were excluded from analysis and epitope number measured using NIS® Element 3D spot detector module. All epitope counts were corrected for non-specific binding by subtraction of mean epitope number (6.74) obtained from three unresponsive pHLA^-^ control cell lines including a B2M mutant, a PRAME^–^ or processing deficient cells.

**Tumor infiltrating lymphocytes isolation**

Fresh tumor biopsies from melanoma patients were commercially sourced (Cambridge Bioscience, UK). Biopsies were dissociated using the Tumor Dissociation Kit and Miltenyi GentleMACS Dissociator (both from Miltenyi Biotec) in accordance with the manufacturer’s protocol. The dissociated tumors were further passed through a 70 µM Miltenyi SmartStrainer (Miltenyi Biotec) to obtain single cell digests. TILs were enriched using a Pan-T Cell Isolation Kit (Miltenyi, Biotec). Live CD8^+^ T cells were FACS sorted on the basis of PD-1 expression using MA900 multi-application cell sorter (Sony Biotechnology, UK).

**In vitro T cell exhaustion model**

Blood from healthy donors was obtained and PBMCs isolated by density gradient centrifugation over Lymphoprep (Axis-Shields). Untouched T cells were purified by immunomagnetic beads using a Pan-T Cell Isolation Kit (Miltenyi Biotec). Induction of T cell exhaustion *in vitro* was adapted from Balkhi *et al*. (Balkhi, Wittmann, Xiong, & Junghans, 2018) In brief, cells were stimulated with Human T-Activator CD3/CD28 Dynabeads™ (ThermoFisher Scientific) and 25 IU IL-2 (Proleukin Novartis). Cells were repeatedly stimulated every 3-4 days and assessed by flow cytometry after each stimulation. Flow cytometry was performed by staining with anti-CD8-APC (BD Biosciences, UK), anti-CD4-PE-Cy7 (BD Biosciences, UK), Zombie Green (viability), anti-PD1-PE, anti-IL7Rα-BV421, and anti-LAG-3-BV785 antibodies (all from Biolegend). After 4 repeated stimulations, T cells were also stained with anti-PD-1 PE and anti-CD8 APC antibodies then FACS sorted on the basis of PD-1 expression using MA900 multi-application cell sorter (Sony Biotechnology Inc.).

**Statistical analysis**

The Wilcoxon test was used to assess *PRAME* expression in log_2_ TPM between sample groups. The number of total somatic mutations per patient were determined and individual patients were stratified according to total number of mutations (low: <500; high: ≥500). Tests were two-sided and were carried out using R (v4.1). The abundance of tissue-infiltrating immune cell populations was determined using MCP-counter version 1.2. (Becht et al., 2016) Transformed PRAME counts per million (TPM) were generated using edgeR (v3.34) (Robinson, McCarthy, & Smyth, 2010) Euclidean distance was applied per patient to generate a distance matrix and complete-linkage clustering was carried out (R ComplexHeatmap v2.3) (Gu, Eils, & Schlesner, 2016).

**SUPPLEMENTARY FIGURES**

**Supplementary Figure 1: High PRAME expression is restricted to tumor and testis. A)** Gene expression analysis of PRAME in normal human tissues compared to primary melanoma tumors. Gene expression of PRAME was analyzed by RNA sequencing in a panel of 40 normal human tissues from the Genotype-Tissue Expression (GTEx) dataset as well as Cutaneous Melanoma primary tumor from The Cancer Genome Atlas (TCGA). **B)** Normal Tissue Expression Analysis of PRAME in Normal Tissues by IHC. Protein expression of PRAME was evaluated by IHC in a panel of 39 normal human tissues from FFPE TMA (tissue microarray), resections and blood smears.


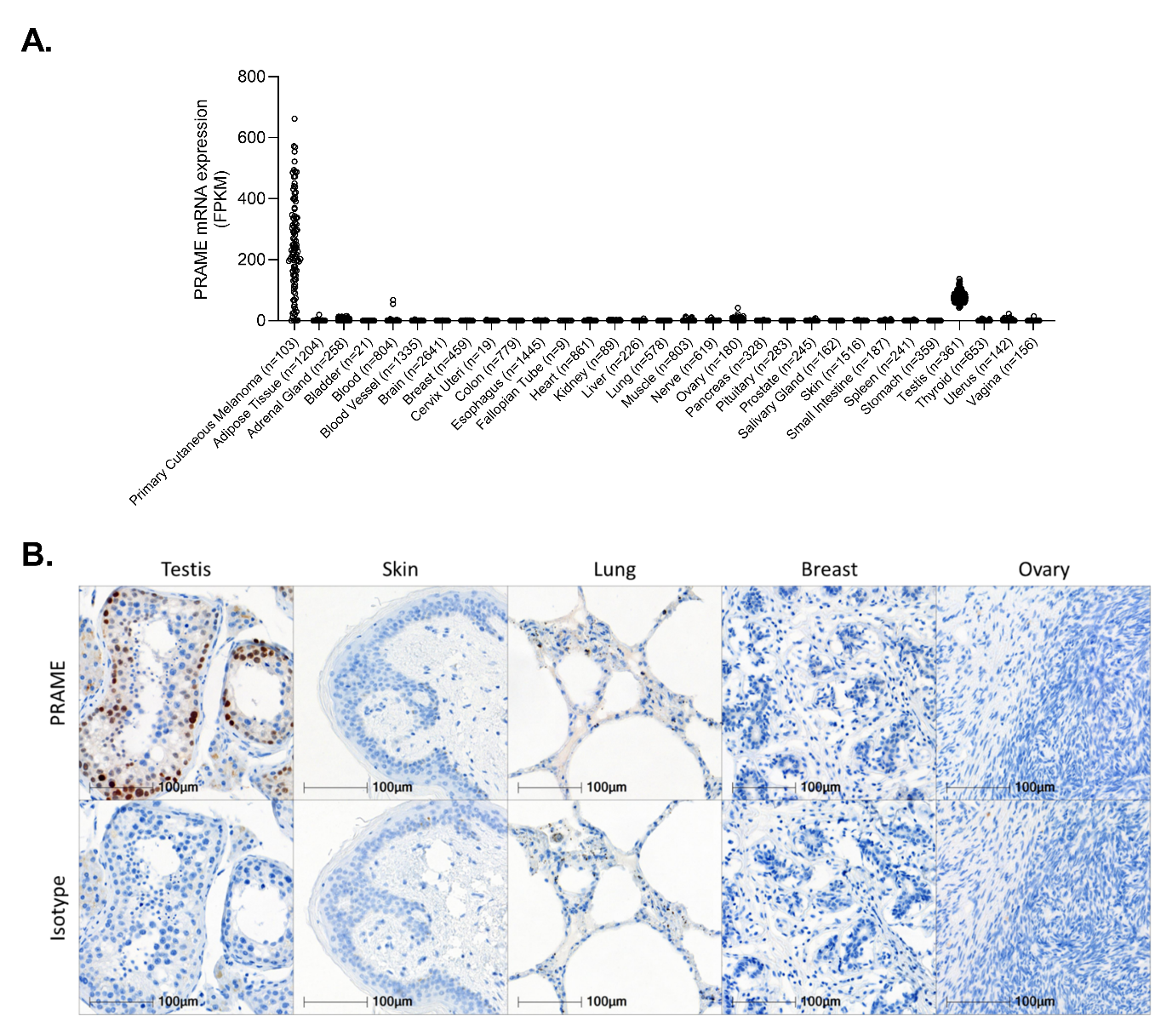


**Supplementary Figure 2:  *PRAME* is widely expressed in lung tumors independently of oncogenic driver mutations, mutation burden and immune infiltration status.** Analyses of *PRAME* expression in individual patient non small cell lung cancer (NSCLC) Adeno **(A)** and squamous **(B)** tumors within the depicted groups. with or without mutations in epithelial growth factor receptor *(EGFR)*, Kelch-like ECH-associated protein 1 (*KEAP1)*, Kirsten-ras small G-protein oncogene *(KRAS)* or serine/threonine kinase-11 (*STK11)* genes, and with distinct overall tumor mutation burden (TMB) and Programmed death-ligand 1 (*PD-L1)* levels. The Wilcoxon test was used to compare *PRAME* expression between sample groups. **(C)** Pathway association analyses after unsupervised clustering of bulk lung tumor RNAseq, comparing *PRAME* expression with presence of infiltrating immune populations assessed by transcript profiling.


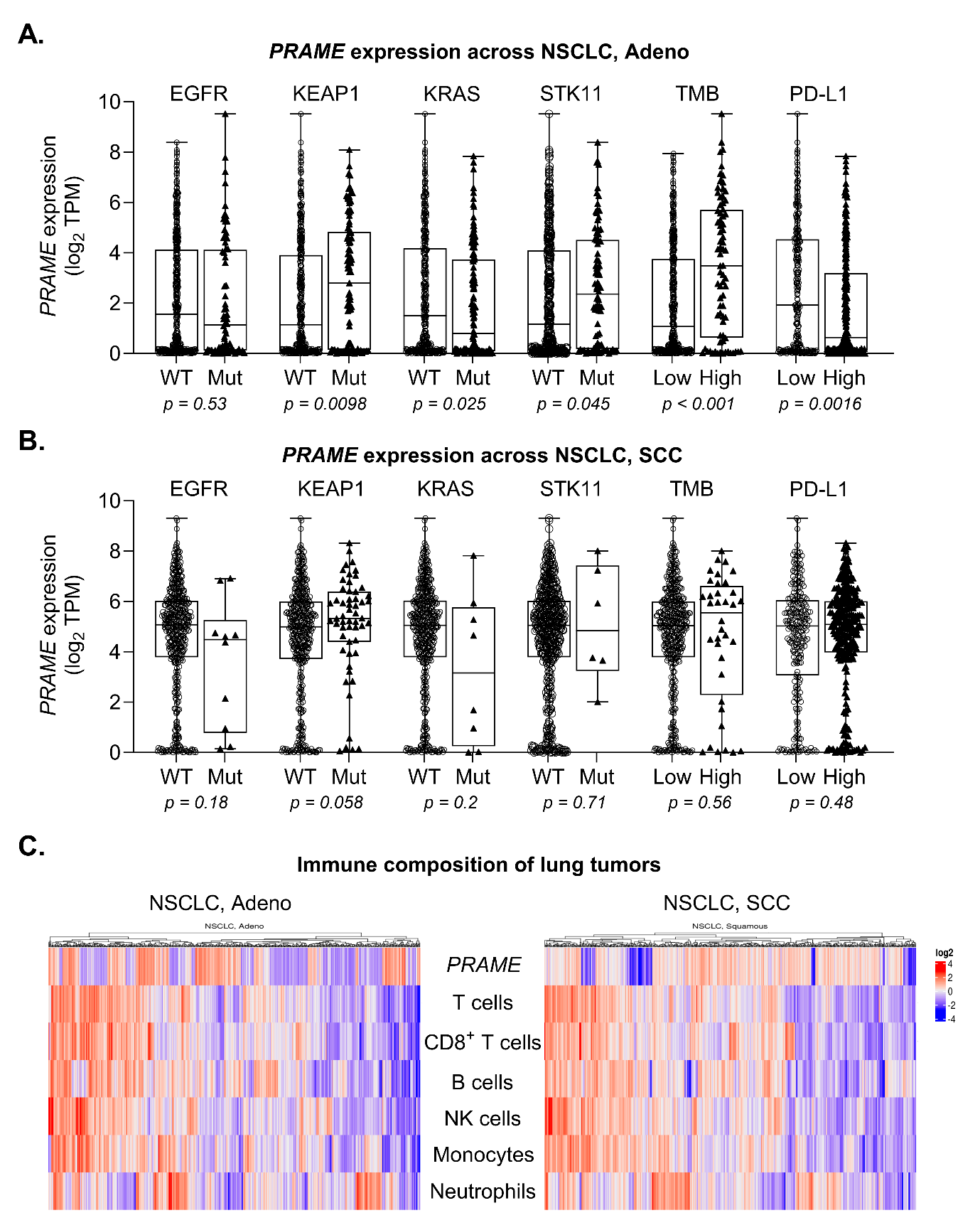


**Supplementary Figure 3**

**Supplementary Figure 3: TCR identification and affinity maturation steps for the PRAME-targeting IMC-F106C ImmTAC molecule. A)** Blood from 26 HLA-A*02:01^+^ healthy donors was screened for the presence of PRAME-specific TCRs. In total, 10 TCRs were found to bind the PRAME-SLL peptide-HLA complex, and four of these (with affinities below 500 µM) were progressed for affinity maturation workflow **(B)**. Three rounds of affinity maturation were applied to the lead WT TCR as well as α and β chain combinations to reach IMC-F106C final sequence.


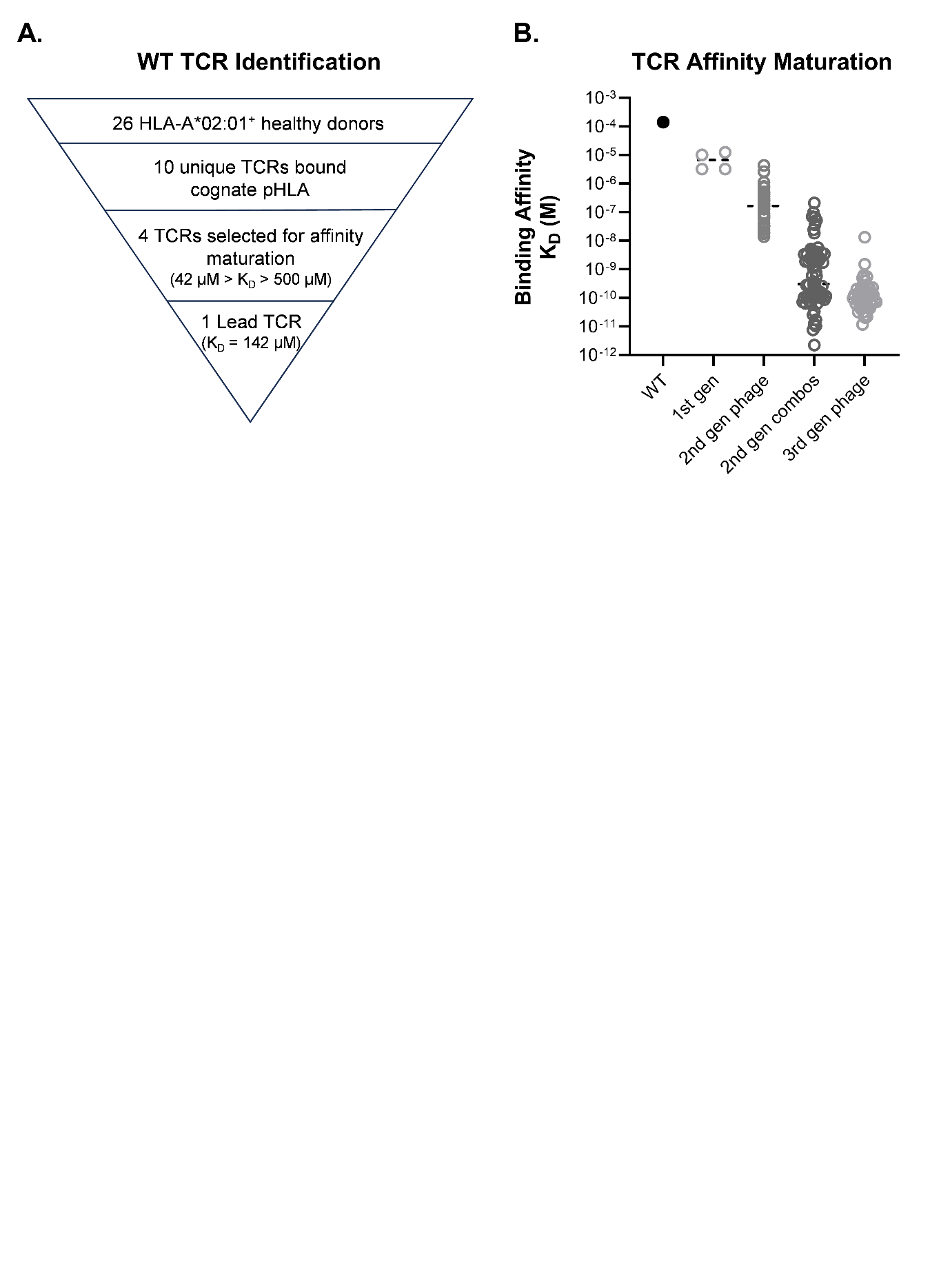


**Supplementary Figure 4: An affinity-enhanced bispecific CD3/TCR ImmTAC® molecule mediates specific T cell activation and redirected killing of melanoma, lung and ovarian tumor cells expressing PRAME (Data from additional PBMC donor).** Human leucocyte antigen (HLA)-relevant PRAME^+^ melanoma **(A)**, non small cell lung cancer (NSCLC) **(B)** and ovarian **(C)** tumor cells were incubated with peripheral blood mononuclear cell (PBMC) effector cells in the presence of increasing concentrations of the PRAME ImmTAC® IMC-F106C. Ovarian TYK-nu (PRAME^–^/HLA-A*02:01^+^) and lung NCI-H1693 (PRAME^+^/HLA-irrelevant) cell lines were included as controls. Effector cells were also cultured with 2 nM IMC-F106C in the absence of target cells, as control. ImmTAC®-mediated T cell activation was determined by IFNγ ELISpot (left panels). $ represents spots too numerous to count by the software and attributed highest counted value. IMC-F106C-mediated T cell killing was determined as % tumor cell cytolysis in xCELLigence impedance-based assays (right panels). D) IMC-F106C-mediated T cell killing data for the PRAME^–^/HLA-A*02:01^+^ TYK-nu cell line, including SLL peptide control. Data were obtained using three healthy PBMC donors and representative data is shown.


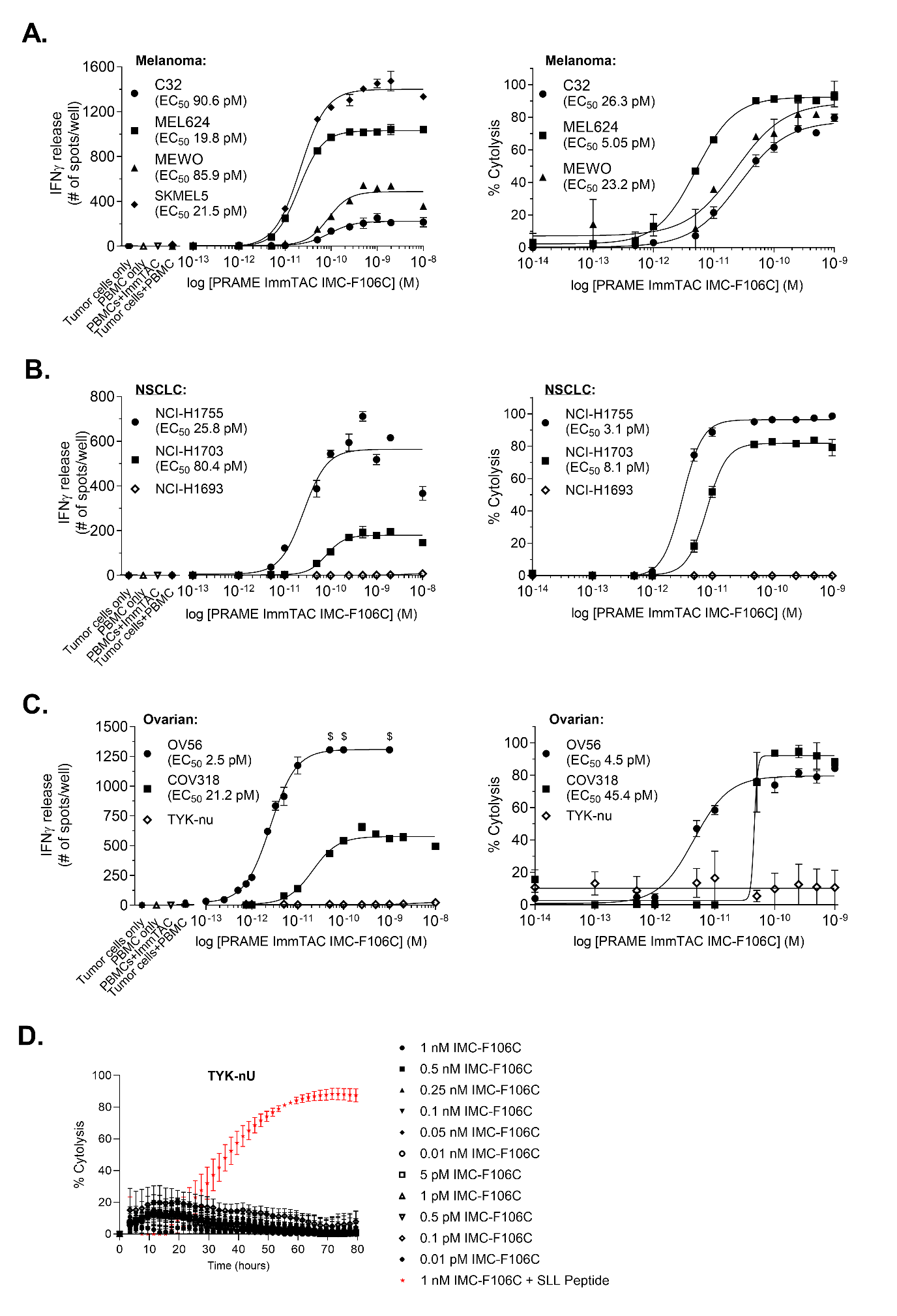


**Supplementary Figure 5: Testing of IMC-F106C ImmTAC® against primary normal cells of skin and lung origin.** Human leucocyte antigen (HLA)-relevant PRAME^–^ melanocytes and bronchial epithelial cells were incubated with PBMC effector cells in the presence of increasing concentrations of PRAME ImmTAC® IMC-F106C. Lung NCI-H1755 (PRAME^+^/HLA-A*02:01^+^) was included as a control. ImmTAC®-mediated T cell activation was determined by IFNγ ELISpot. Data shown were obtained using one representative healthy PBMC donor of n=3.


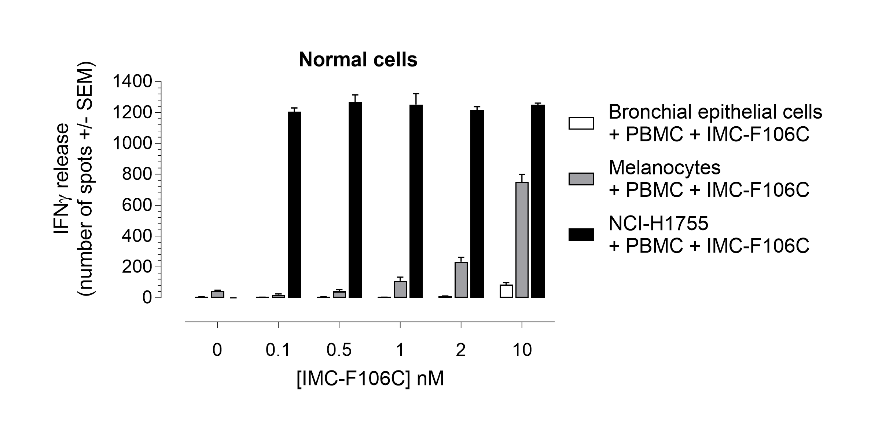


**Supplementary Figure 6: IMC-F106C ImmTAC® induces PRAME pHLA-dependent apoptosis in a tumor organoid model.** Caspase activity following 48 hours co-culture of PRAME^+^ and PRAME^–^ patient-derived organoids with human leucocyte antigen (HLA)-matched healthy peripheral blood mononuclear cells (PBMC) in the presence of increasing concentrations of PRAME-specific ImmTAC® IMC-F106C. Bars represent mean ±SD of three PBMC donors.


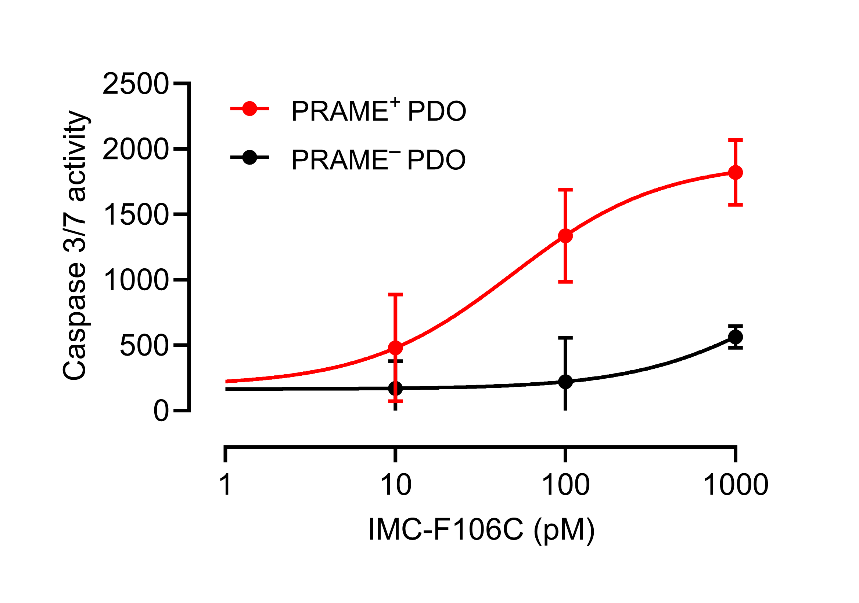


**Supplementary Figure 7: ImmTAC®-mediated redirection of chronically stimulated PD-1^+^ T cells is reduced against PD-L1^+^ tumours, an effect alleviated by anti-PD1 antibody. (A)** T cell phenotype following exhaustion *in vitro*. Repeated stimulation (every 3-4 days) resulted in gradual loss of expression of the interleukin 7 receptor alpha (IL-7Rα) memory marker and upregulation of the exhaustion markers programmed cell death protein 1 (PD-1) and lymphocyte activation gene 3 (LAG-3). Histograms show CD8 T cell phenotype (similar data were observed for CD4 T cells, data not shown). **(B)** Exhausted PD-1^+^ FACS sorted T cells (four stimulations, right panel) and non-exhausted PD-1^–^ T cells (one stimulation, left panel) were redirected to kill PD-L1^+^ (PD-L1 transduced MEL624 cells) and PD-L1^–^ tumours (MEL624 cells) in the presence of ImmTAC® alone (white box plots) or ImmTAC® with anti-PD-1 antibody (grey box plots). Cumulative data is shown from four donors.

***
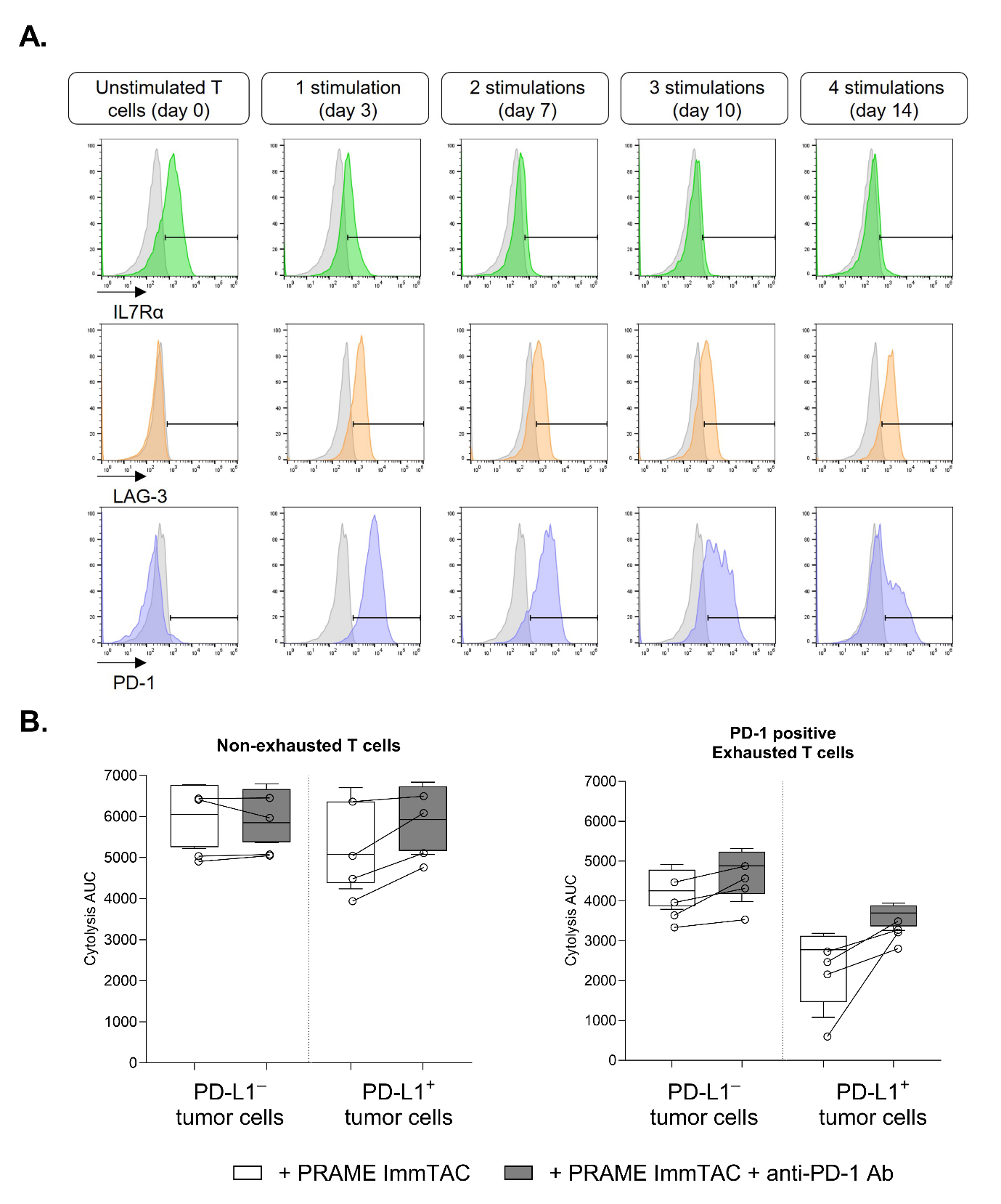
***

**Supplementary Table S1:** Cancer cell lines used for T cell activation and redirected killing assays and epitope counting, and respective tumor origin.

| Cell line | Tumor type |
| --- | --- |
| NCI-H1755 | NSCLC adenocarcinoma |
| NCI-H1703 | NSCLC squamous |
| NCI-H1693 | NSCLC adenocarcinoma |
| OV56 | Ovarian serous carcinoma |
| COV318 | Ovarian serous carcinoma |
| TYK-Nu | Ovarian serous carcinoma |
| NCI-H441 | NSCLC adenocarcinoma |
| MEL624 / 624MEL | Cutaneous melanoma |
| CHP212 | Neuroblastoma |
| Colo699 | Lung adenocarcinoma |
| MEWO | Cutaneous melanoma |
| OVCAR3 | Ovarian serous carcinoma |
| Colo818 | Cutaneous melanoma |
| C32 | Cutaneous melanoma |
| A375 | Cutaneous melanoma |
| NCIH2023 | Lung adenocarcinoma |
| SKMEL5 | Cutaneous melanoma |
| SW982 | Synovial sarcoma |
| WM115 | Cutaneous melanoma |
| WM2664 | Cutaneous melanoma |

**Supplementary Table 3:** Summary of clones generated during two rounds of PRAME peptide sequence CRISPR editing of the MEL624 cell line (wt – wild type; bp – base pairs).

| **Cell line** | **Allele 1** | **Allele 2** | **Allele 3** | **Target peptide** |
| --- | --- | --- | --- | --- |
| **MEL624 (parental)** | wt | wt | wt | SLLQHLIGL x3 |
| **MEL624 KO5** | wt | -2bp | -59bp +8bp | SLLQHLIGL/SLLQHLMAE/deleted |
| **MEL624 A08** | +1bp | -2bp | -59bp +8bp | unknown/SLLQHLMAE/deleted |

**References:**

Balkhi, M. Y., Wittmann, G., Xiong, F., & Junghans, R. P. (2018). YY1 Upregulates Checkpoint Receptors and Downregulates Type I Cytokines in Exhausted, Chronically Stimulated Human T Cells. *IScience*, *2*, 105–122. doi: 10.1016/j.isci.2018.03.009

Becht, E., Giraldo, N. A., Lacroix, L., Buttard, B., Elarouci, N., Petitprez, F., … Reyniès, A. de. (2016). Estimating the population abundance of tissue-infiltrating immune and stromal cell populations using gene expression. *Genome Biology*, *17*(1), 218. doi: 10.1186/s13059-016-1070-5

Gu, Z., Eils, R., & Schlesner, M. (2016). Complex heatmaps reveal patterns and correlations in multidimensional genomic data. *Bioinformatics*, *32*(18), 2847–2849. doi: 10.1093/bioinformatics/btw313

Li, Y., Moysey, R., Molloy, P. E., Vuidepot, A.-L., Mahon, T., Baston, E., … Boulter, J. M. (2005). Directed evolution of human T-cell receptors with picomolar affinities by phage display. *Nature Biotechnology*, *23*(3), 349–354. doi: 10.1038/nbt1070

Liddy, N., Bossi, G., Adams, K. J., Lissina, A., Mahon, T. M., Hassan, N. J., … Jakobsen, B. K. (2012). Monoclonal TCR-redirected tumor cell killing. *Nature Medicine*, *18*(6), 980–987. doi: 10.1038/nm.2764

McCormack, E., Adams, K. J., Hassan, N. J., Kotian, A., Lissin, N. M., Sami, M., … Jakobsen, B. K. (2013). Bi-specific TCR-anti CD3 redirected T-cell targeting of NY-ESO-1- and LAGE-1-positive tumors. *Cancer Immunology, Immunotherapy*, *62*(4), 773–785. doi: 10.1007/s00262-012-1384-4

Robinson, M. D., McCarthy, D. J., & Smyth, G. K. (2010). edgeR: a Bioconductor package for differential expression analysis of digital gene expression data. *Bioinformatics*, *26*(1), 139–140. doi: 10.1093/bioinformatics/btp616
